# Engineering an *Escherichia coli* strain for enhanced production of flavonoids derived from pinocembrin

**DOI:** 10.1186/s12934-024-02582-z

**Published:** 2024-11-19

**Authors:** Erik K. R. Hanko, Christopher J. Robinson, Sahara Bhanot, Adrian J. Jervis, Nigel S. Scrutton

**Affiliations:** Manchester Institute of Biotechnology, Faculty of Science and Engineering, University of Manchester, 131 Princess Street, Manchester, M1 7DN UK

**Keywords:** Pinocembrin, Flavonoid, Chrysin, Pinostrobin, Pinobanksin, Galangin, *Escherichia coli*

## Abstract

**Background:**

Flavonoids are a structurally diverse group of secondary metabolites, predominantly produced by plants, which include a range of compounds with pharmacological importance. Pinocembrin is a key branch point intermediate in the biosynthesis of a wide range of flavonoid subclasses. However, replicating the biosynthesis of these structurally diverse molecules in heterologous microbial cell factories has encountered challenges, in particular the modest pinocembrin titres achieved to date. In this study, we combined genome engineering and enzyme candidate screening to significantly enhance the production of pinocembrin and its derivatives, including chrysin, pinostrobin, pinobanksin, and galangin, in *Escherichia coli*.

**Results:**

By implementing a combination of established strain engineering strategies aimed at enhancing the supply of the building blocks phenylalanine and malonyl-CoA, we constructed an *E. coli* chassis capable of accumulating 353 ± 19 mg/L pinocembrin from glycerol, without the need for precursor supplementation or the fatty acid biosynthesis inhibitor cerulenin. This chassis was subsequently employed for the production of chrysin, pinostrobin, pinobanksin, and galangin. Through an enzyme candidate screening process involving eight type-1 and five type-2 flavone synthases (FNS), we identified *Petroselinum crispum* FNSI as the top candidate, producing 82 ± 5 mg/L chrysin. Similarly, from a panel of five flavonoid 7-*O*-methyltransferases (7-OMT), we found pinocembrin 7-OMT from *Eucalyptus nitida* to yield 153 ± 10 mg/L pinostrobin. To produce pinobanksin, we screened seven enzyme candidates exhibiting flavanone 3-hydroxylase (F3H) or F3H/flavonol synthase (FLS) activity, with the bifunctional F3H/FLS enzyme from *Glycine max* being the top performer, achieving a pinobanksin titre of 12.6 ± 1.8 mg/L. Lastly, by utilising a combinatorial library of plasmids encoding *G. max* F3H and *Citrus unshiu* FLS, we obtained a maximum galangin titre of 18.2 ± 5.3 mg/L.

**Conclusion:**

Through the integration of microbial chassis engineering and screening of enzyme candidates, we considerably increased the production levels of microbially synthesised pinocembrin, chrysin, pinostrobin, pinobanksin, and galangin. With the introduction of additional chassis modifications geared towards improving cofactor supply and regeneration, as well as alleviating potential toxic effects of intermediates and end products, we anticipate further enhancements in the yields of these pinocembrin derivatives, potentially enabling greater diversification in microbial hosts.

**Supplementary Information:**

The online version contains supplementary material available at 10.1186/s12934-024-02582-z.

## Background

**Fig. 1:**
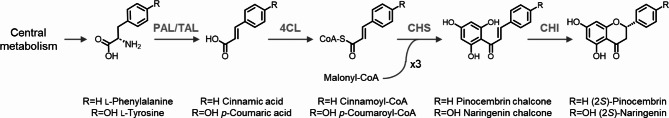
The (2*S*)-flavanone biosynthesis pathway in plants. Enzyme abbreviations: PAL, phenylalanine ammonia-lyase; TAL, tyrosine ammonia-lyase; 4CL, 4-coumarate-CoA ligase; CHS, chalcone synthase; CHI, chalcone isomerase

Flavonoids are a class of structurally diverse secondary metabolites that are widespread throughout the plant kingdom. Within plants, they play various roles, ranging from contributing to flower pigmentation to providing protection against UV radiation and regulating various physiological processes [1, 2]. These compounds are derived from the phenylpropanoid pathway, which generates the (hydroxy)cinnamoyl-CoA intermediate (Fig. 1). Subsequently, chalcone synthase (CHS) and chalcone isomerase (CHI) catalyse the synthesis of the (2*S*)-flavanone product, requiring three units of malonyl-CoA per flavanone molecule. The (2*S*)-flavanone backbone itself acts as a key branch point for structural diversification, serving as the substrate for the synthesis of other flavonoid subclasses, including flavones, isoflavones, dihydroflavonols, flavonols, and anthocyanins.

(2*S*)-Pinocembrin is one of the key branch point flavanones, serving as the precursor for a range of flavonoid products, including chrysin, pinostrobin, pinobanksin, and galangin. In nature, these compounds collectively constitute a considerable portion of the flavonoids present in poplar buds [3]. As a result, they are commonly found in poplar-based honey and propolis, the latter also known as bee glue [4, 5]. They contribute to the bioactive properties of these products, including their anti-inflammatory, antioxidant, and antimicrobial effects, thereby highlighting the potential of pinocembrin and its derivatives as promising drug candidates for clinical applications [6–10].

Due to their pharmacological potential, the heterologous production of flavonoids like pinocembrin has been extensively investigated. For example, using recombinant *Escherichia coli*, a pinocembrin titre of 710 mg/L has been previously achieved [11]. This engineered strain harboured a heterologous flavanone biosynthesis pathway comprising 4-coumarate-CoA ligase (4CL), CHS, and CHI, and was supplemented with cinnamic acid as the phenylpropanoid precursor and the fatty acid biosynthesis inhibitor cerulenin. In the absence of the costly cerulenin, a titre of 29 mg/L was obtained [11]. Another approach involved overexpressing the same flavanone biosynthesis pathway along with acetyl-CoA carboxylase (ACC) from *Photorhabdus luminescens* and the endogenous acetyl-CoA synthetase, facilitating malonyl-CoA synthesis from acetate and resulting in a pinocembrin titre of 429 mg/L [12]. Although this strategy significantly enhanced acetate assimilation, it still required cinnamic acid supplementation.

In an attempt to eliminate the need for precursor supplementation, Wu and colleagues incorporated phenylalanine ammonia-lyase (PAL) into the flavanone biosynthesis pathway (Fig. 1), converting phenylalanine into cinnamic acid [13]. This enabled the direct production of pinocembrin from central metabolism. By boosting phenylalanine synthesis and incorporating a malonate assimilation pathway, a pinocembrin titre of 432 mg/L was achieved in glucose fed-batch fermentation [13]. In *Saccharomyces cerevisiae*, pinocembrin production has been less successful, with titres reaching only 80 mg/L [14]. Despite the relatively high titres of pinocembrin that can be obtained in *E. coli*, there has been little focus on the structural modification of the pinocembrin scaffold. Only low titres of chrysin (9.4 mg/L) and galangin (1.1 mg/L) have been reported [15], substantially limiting their utility in pharmacological testing.

In this study, we integrate established strain engineering strategies to develop an *E. coli* chassis capable of accumulating significant quantities of pinocembrin from glycerol without requiring precursor supplementation or the fatty acid biosynthesis inhibitor cerulenin. To enable the one-step conversion of pinocembrin into chrysin, pinostrobin, and pinobanksin, we screen several enzyme candidates with flavone synthase, flavonoid 7-*O*-methyltransferase, flavanone-3-hydroxylase, and flavanone-3-hydroxylase/flavonol synthase activities, respectively. Additionally, to optimise the two-step conversion of pinocembrin into galangin via pinobanksin, we test a combinatorial library of plasmids expressing *Glycine max* flavanone-3-hydroxylase and *Citrus unshiu* flavonol synthase. This study lays the groundwork for achieving further structural diversification of pinocembrin derivatives in microbial cell factories.

## Results and discussion

### Construction of a pinocembrin production chassis

**Fig. 2:**
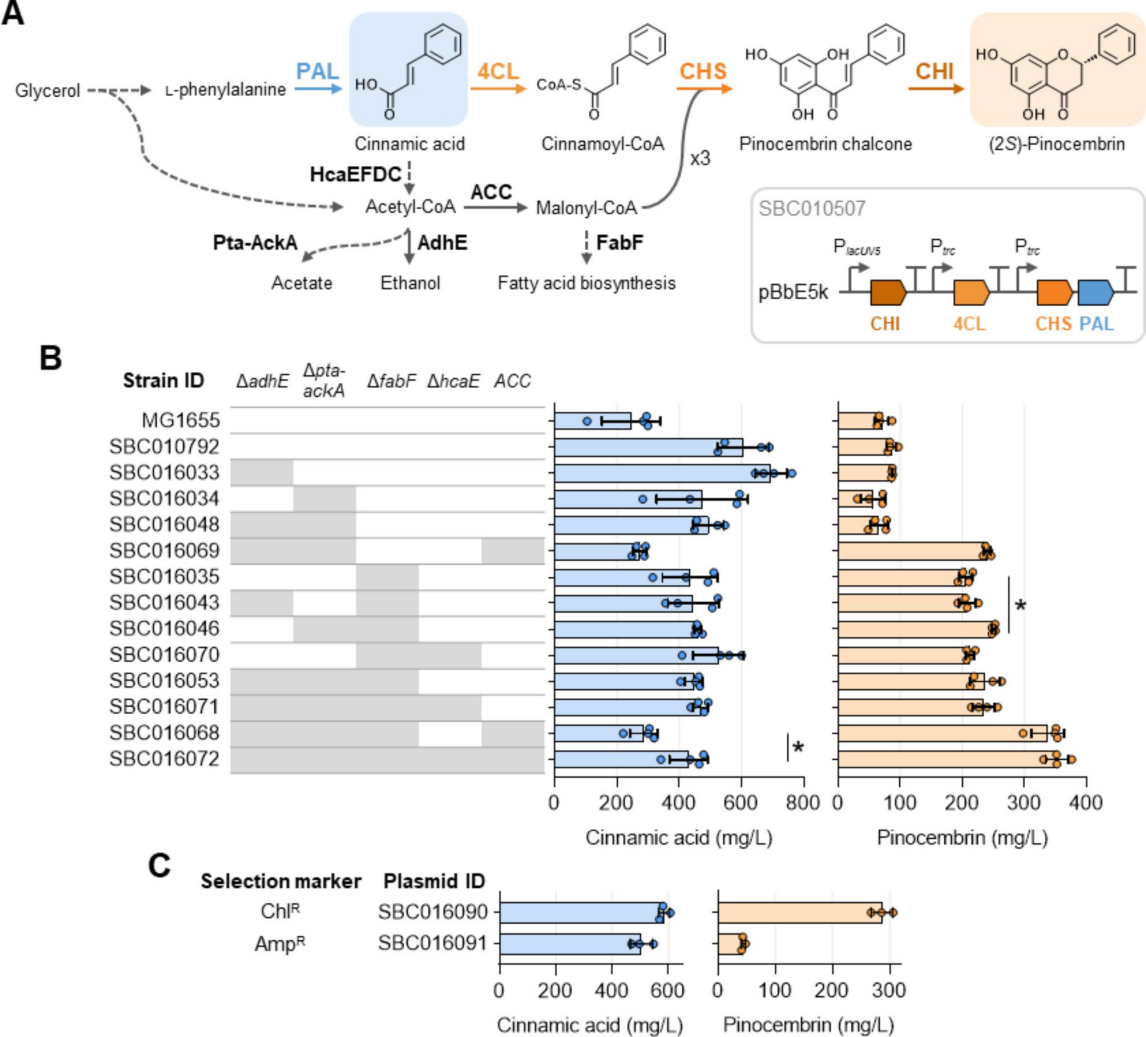
Construction of an *E. coli* pinocembrin chassis. **A** Biosynthesis of (2*S*)-pinocembrin from l-phenylalanine in *E. coli*. The optimised pinocembrin pathway, encoded by plasmid SBC010507, comprises PAL from *Arabidopsis thaliana*, 4CL from *Glycine max*, CHS from *Camellia sinensis*, and CHI from *A. thaliana*. Other enzyme abbreviations: ACC, acetyl-CoA carboxylase; AdhE, bifunctional aldehyde-alcohol dehydrogenase; FabF, 3-oxoacyl-[acyl-carrier-protein] synthase 2; HcaEFDC, cinnamate dioxygenase; Pta-AckA, phosphate acetyltransferase-acetate kinase. Solid arrows indicate reactions catalysed by a single enzyme or enzyme complex. **B** Cinnamic acid (light blue) and pinocembrin (light orange) titres in different *E. coli* strains carrying plasmid SBC010507. Genome modifications are indicated by shaded boxes. Expression of genes was induced by addition of IPTG. Data are presented as mean ± standard deviation, *n* = 4, *p < 0.01, unpaired two-tailed *t*-test. **C** Cinnamic acid (light blue) and pinocembrin (light orange) titres in strain SBC016072 carrying either plasmid SBC016090 (chloramphenicol resistance marker) or SBC016091 (ampicillin resistance marker). Expression of genes was induced by addition of IPTG. Data are presented as mean ± standard deviation, *n* = 3. Source data are available in the Source Data file

To maximise the bioproduction of chrysin, pinostrobin, pinobanksin, and galangin, our initial focus was on building a base strain capable of accumulating sufficient amounts of the intermediate flavanone (2*S*)-pinocembrin. For the production of pinocembrin, we employed a previously established biosynthesis pathway (plasmid SBC010507, Fig. 2A), comprised of phenylalanine ammonia-lyase (PAL) and chalcone isomerase (CHI) from *Arabidopsis thaliana* (thale cress), in conjunction with 4-coumarate-CoA ligase (4CL) from *Glycine max* (soybean), and chalcone synthase (CHS) from *Camellia sinensis* (tea) [16]. This version of the pinocembrin biosynthesis pathway was obtained through screening of enzyme candidate libraries and iterative optimisation of genetic construct design [16, 17]. It yielded 198 mg/L pinocembrin in the *E. coli* wildtype strain MG1655 when supplemented with the pathway substrate l-phenylalanine and the fatty acid biosynthesis inhibitor cerulenin [16]. However, due to the economic impracticality of l-phenylalanine and cerulenin supplementation at a large scale, in this study we aimed to implement a series of host strain genome modifications that could enhance pinocembrin production without the need for additional media supplements.

Drawing from various successful studies on flavonoid production in *E. coli*, including the flavanones naringenin and pinocembrin, we adopted a combination of three strategies to enhance the levels of malonyl-CoA available for pinocembrin biosynthesis in strain MG1655. The first strategy involved eliminating non-essential pathways that compete for acetyl-CoA (Fig. 2A). Since acetyl-CoA serves as the direct precursor of malonyl-CoA, essential for CHS to build the chalcone intermediate, enhancing acetyl-CoA levels has been shown to bolster flavanone production [18, 19]. We deleted the two-step Pta-AckA pathway, responsible for converting acetyl-CoA into acetate, and AdhE, which catalyses the two-step reduction of acetyl-CoA into ethanol. The second strategy focused on overexpressing the heterologous acetyl-CoA carboxylase (ACC) from *Corynebacterium glutamicum*, denoted CgACC, to augment the conversion of acetyl-CoA to malonyl-CoA. Overexpression of the ACC subunits *accBC* and *accD1* in *E. coli* has been reported to increase the intracellular concentration of malonyl-CoA by threefold [20]. Here, we additionally integrated the small ε-subunit, AccE, which has been found to be part of the CgACC complex [21]. The third strategy aimed to mimic the effect of cerulenin supplementation by limiting the flux toward fatty acid biosynthesis, the sole fate of malonyl-CoA in *E. coli*. To achieve this, we deleted *fabF*, which encodes the non-essential β-ketoacyl-[acyl carrier protein] synthase (KAS) 2, involved in fatty acid elongation. FabF is one of two KAS enzymes whose function is inhibited by cerulenin [22]. In this study, all genome modifications were conducted in strain SBC010792, a derivative of MG1655 with improved flux toward l-phenylalanine production. This strain had an 8.7-kbp sequence inserted into the genome at the *lacZ* locus, which included recoded copies of the *E. coli ppsA*, *aroF*, and *pheA* genes, with mutations that relieve feedback inhibition by phenylalanine [23, 24], under the control of the IPTG-inducible *lacUV5* promoter.

Deletion of *adhE*, either alone or in combination with other genes, generally did not impact pinocembrin production (Fig. 2B), most likely due to the ethanol-forming pathway being inactive under aerobic growth conditions [25]. Similarly, deletion of *pta-ackA*, encoding one of the two known acetate-forming pathways, only exhibited a slight positive effect on pinocembrin biosynthesis in the FabF-deficient background (strain SBC016046 vs. SBC016035). Previous studies have observed that acetate does not accumulate when glycerol is used as the carbon source, hence, eliminating the Pta-AckA pathway may have little or no effect on acetyl-CoA levels under our growth conditions [26, 27].

The most dramatic increase in pinocembrin titres was observed upon deleting *fabF*. This single modification led to a 2.4-fold rise in pinocembrin levels in the phenylalanine chassis strain SBC010792, indicating a substantial redirection of malonyl-CoA from fatty acid to flavonoid biosynthesis. A similar fold-increase in pinocembrin titres was previously observed upon deleting *fabF* in the parent strain MG1655 [17]. Interestingly, previous studies have reported that overexpression of *fabF* also enhances intracellular malonyl-CoA supply [20], possibly due to elevated levels of FabF obstructing the access of the other two KAS enzymes, FabB and FabH, to the malonyl-CoA-acyl carrier protein transacylase, FabD. This interference has been proposed to stall both the initiation of fatty acid biosynthesis and fatty acid elongation [28]. However, since the combined overexpression of *fabF* and *acc* has been shown to negate the positive effect on malonyl-CoA supply resulting from the individual overexpression of these genes [20], we decided to proceed with the FabF-deficient strain.

The integration of CgACC had a significant positive effect on pinocembrin production in the strain where the pathways consuming acetyl-CoA were deleted, resulting in a pinocembrin titre of 240 mg/L, corresponding to a 3.7-fold higher pinocembrin titre in strain SBC016069 compared to its counterpart lacking CgACC. This increase aligns with the previously observed threefold rise in malonyl-CoA availability when *accBC* and *accD1* were overexpressed [20]. Furthermore, the deletion of *fabF* in strain SBC016068 further enhanced the pinocembrin titre to 338 mg/L, demonstrating that the combination of CgACC overexpression and *fabF* deletion improved malonyl-CoA titres by more than fivefold.

The final strain modification involved eliminating a cinnamic acid degradation pathway, responsible for converting the phenylpropanoid precursor cinnamic acid into acetyl-CoA [29]. Catabolism of cinnamic acid is initiated by cinnamate dioxygenase. Through the deletion of its large subunit, HcaE, we have previously constructed an *E. coli* strain that did not consume exogenously supplemented cinnamic acid [30]. While cinnamic acid availability did not seem to limit pinocembrin production, we sought to eliminate this catabolic pathway to reduce the metabolic resources needed to maintain this diversion. Surprisingly, deletion of *hcaE* significantly boosted cinnamate accumulation only when combined with CgACC overexpression. This suggests that, under our growth conditions, production of cinnamate proceeds at a higher rate than its degradation, possibly due to low or no expression of the cinnamic acid degradation pathway. In the final strain, SBC016072, pinocembrin titres reached 353 ± 19 mg/L, and this strain reached a final cell density similar to that of the wildtype strain MG1655 (Supplementary Figure S1). This was despite the fact that the *fabF* deletion alone (SBC016035) reduced the final cell density, indicating that the combined strain modifications alleviated this negative effect on cell growth. Given that *hcaE* deletion had a slight, though not statistically significant, positive effect on pinocembrin biosynthesis, we proceeded with strain SBC016072 as the chassis for the bioproduction of chrysin, pinostrobin, pinobanksin, and galangin.

### Production of chrysin

To enable compatibility with the different downstream pathways utilising vectors with distinct antibiotic resistance markers, we replaced the kanamycin selection marker on pinocembrin pathway plasmid SBC010507 with ampicillin and chloramphenicol resistance markers. Subsequently, we quantified the pinocembrin titres in strain SBC016072 carrying these three vectors. Interestingly, while the chloramphenicol-based vector led to increased cinnamate levels and only a slight decrease in pinocembrin titres (287 mg/L) compared to the kanamycin-based counterpart, the use of the ampicillin selection marker led to an almost tenfold reduction in pinocembrin titres (44 mg/L), while the cinnamate levels remained largely unchanged (Fig. 2C). A similar observation was previously reported for pinocembrin production using a non-optimised biosynthesis pathway, as well as for bio-alkane production in *E. coli* [16, 31]. In both cases, substituting the kanamycin selection marker with an ampicillin marker in pBbE-based vectors resulted in a significant reduction in product titres. Consequently, downstream pathways with chloramphenicol and ampicillin selection markers were co-transformed with the original pinocembrin pathway plasmid, SBC010507, whereas kanamycin-based downstream pathways were co-transformed with plasmid SBC016090, which utilises the chloramphenicol marker to maintain the pinocembrin biosynthesis pathway.

**Fig. 3:**
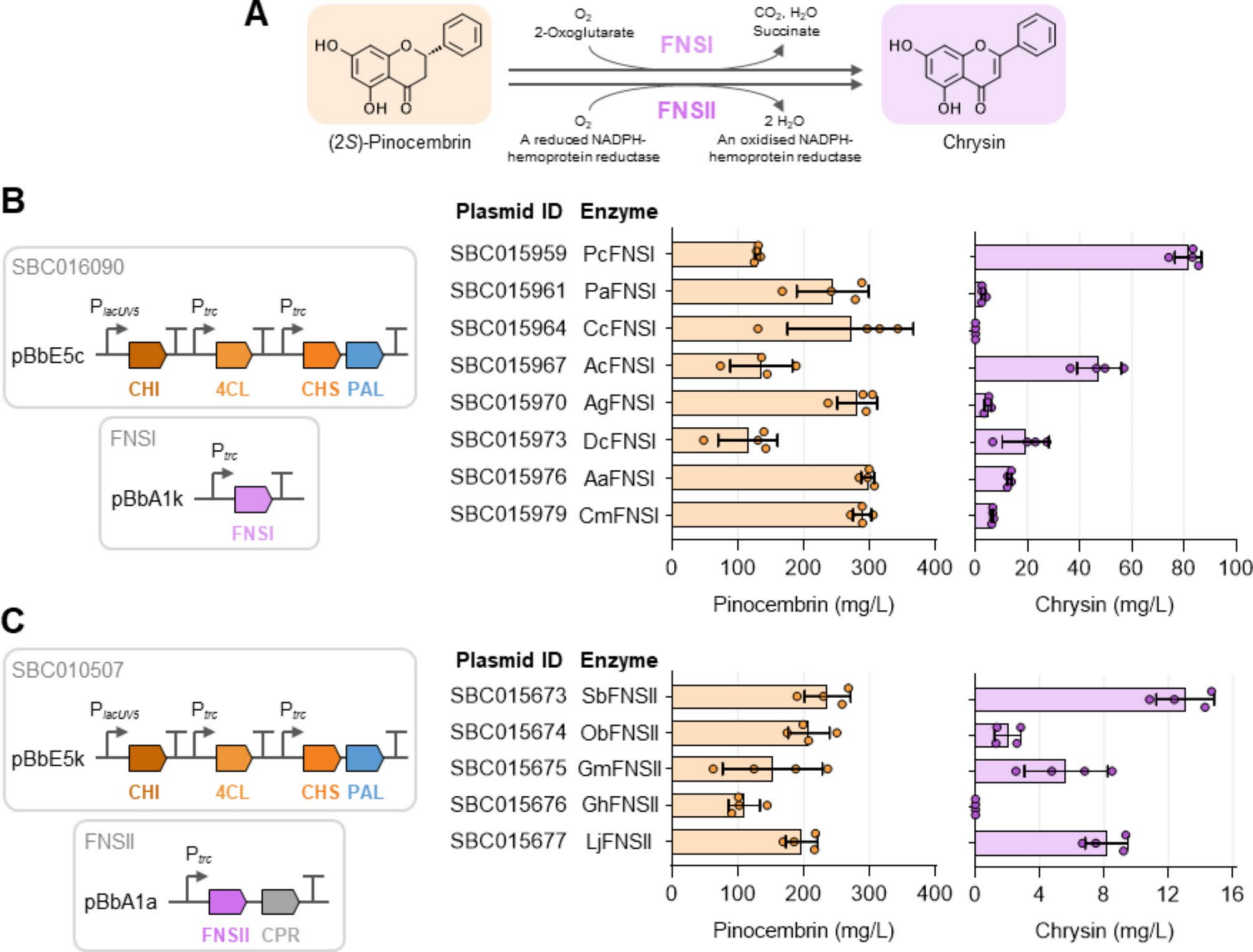
Biosynthesis of chrysin using the *E. coli* pinocembrin chassis. **A** The one-step conversion of (2*S*)-pinocembrin into chrysin is catalysed by type-I or type-II flavone synthase (FNS), with cytochrome P450 reductase (CPR) being required for the catalytic activity of FNSII enzymes. **B** Production of chrysin in strain SBC016072 using a two-plasmid system. Plasmid SBC016090 encodes the optimised pinocembrin pathway, while the second plasmid encodes a library of different FNSI candidate enzymes. Pinocembrin (light orange) and chrysin (light purple) titres are presented for the individual strains. Organism abbreviations: Pc, *Petroselinum crispum*; Pa, *Plagiochasma appendiculatum*; Cc, *Cuminum cyminum*; Ac, *Aethusa cynapium*; Ag, *Apium graveolens*; Dc, *Daucus carota*; Aa, *Angelica archangelica*; Cm, *Conium maculatum*. New enzyme abbreviation: F3H, flavanone 3-hydroxylase. **C** Production of chrysin in strain SBC016072 using a two-plasmid system. Plasmid SBC010507 encodes the optimised pinocembrin pathway, while the second plasmid encodes a library of different FNSII candidate enzymes in conjunction with *Arabidopsis thaliana* CPR. Pinocembrin (light orange) and chrysin (light purple) titres are presented for the individual strains. Organism abbreviations: Sb, *Scutellaria baicalensis*; Ob, *Ocimum basilicum*; Gm, *Glycine max*; Gh, *Gerbera hybrida*; Lj, *Lonicera japonica*. Strains with FNSII enzymes were supplemented with 5-aminolevulinic acid. Expression of genes was induced by addition of IPTG. Data are presented as mean ± standard deviation, *n* = 4. Source data are available in the Source Data file

The oxidation of (2*S*)-pinocembrin, catalysed by flavone synthase (FNS), results in the formation of the flavone chrysin (Fig. 3A). Chrysin is naturally present in various medicinal plants and fruits, and has been reported to exhibit anticancer, anti-inflammatory, and neuroprotective effects [7, 32]. However, its heterologous production in *E. coli* and yeast has been challenging, yielding titres in the low milligram range [14, 15, 33, 34]. One of the primary obstacles in this endeavour has been the limited production of pinocembrin in these hosts. In a recent study, we evaluated two types of FNS enzymes for catalysing the conversion of pinocembrin into chrysin [34]. FNSI enzymes are soluble dioxygenases, whereas FNSII proteins are membrane-bound cytochrome P450 monooxygenases, which require a NADPH-cytochrome P450 reductase (CPR) partner for their activity.

Using the newly constructed pinocembrin chassis, we tested a library of eight FNSI and five FNSII enzyme candidates. The FNSI genes were expressed from a pBbA1k-based vector and hence co-transformed with plasmid SBC016090 (Fig. 3B). Except for *Cuminum cyminum* (cumin) FNSI, all enzyme candidates produced detectable amounts of chrysin. The highest chrysin titre of 82 ± 5 mg/L was obtained using *Petroselinum crispum* (parsley) FNSI, surpassing the chrysin titres previously reported in *E. coli* and yeast. However, it is noteworthy that even in the best-performing strain, 130 mg/L of pinocembrin remained unconverted.

For the production of chrysin using FNSII enzymes, we tested two culture conditions. The strains were grown both with and without 5-aminolevulinic acid (5-ALA), a key intermediate in the biosynthesis of the heme cofactor, which is required for FNSII activity [35]. Except for *Gerbera hybrida* (Transvaal daisy) FNSII, all enzyme candidates yielded detectable amounts of chrysin under both conditions (Fig. 3C, Supplementary Figure S2). However, chrysin titres were notably higher in the presence of 5-ALA. For instance, while the best-performing enzyme candidate, *Scutellaria baicalensis* (Baikal skullcap) FNSII, produced a chrysin titre of 13 mg/L with 5-ALA supplementation, it yielded five times less in its absence. Interestingly, in this enzyme screen, *P. crispum* FNSI outperformed all FNSII enzyme candidates, contrary to previous findings where *G. max* FNSII achieved the highest chrysin titre [34]. This difference in performance might be attributed to the different *E. coli* strains used (MG1655 vs. NEB5α), different antibiotic selection markers (kanamycin vs. ampicillin), or the source of the phenylalanine substrate (endogenously produced vs. supplementation).

### Production of pinostrobin

**Fig. 4:**
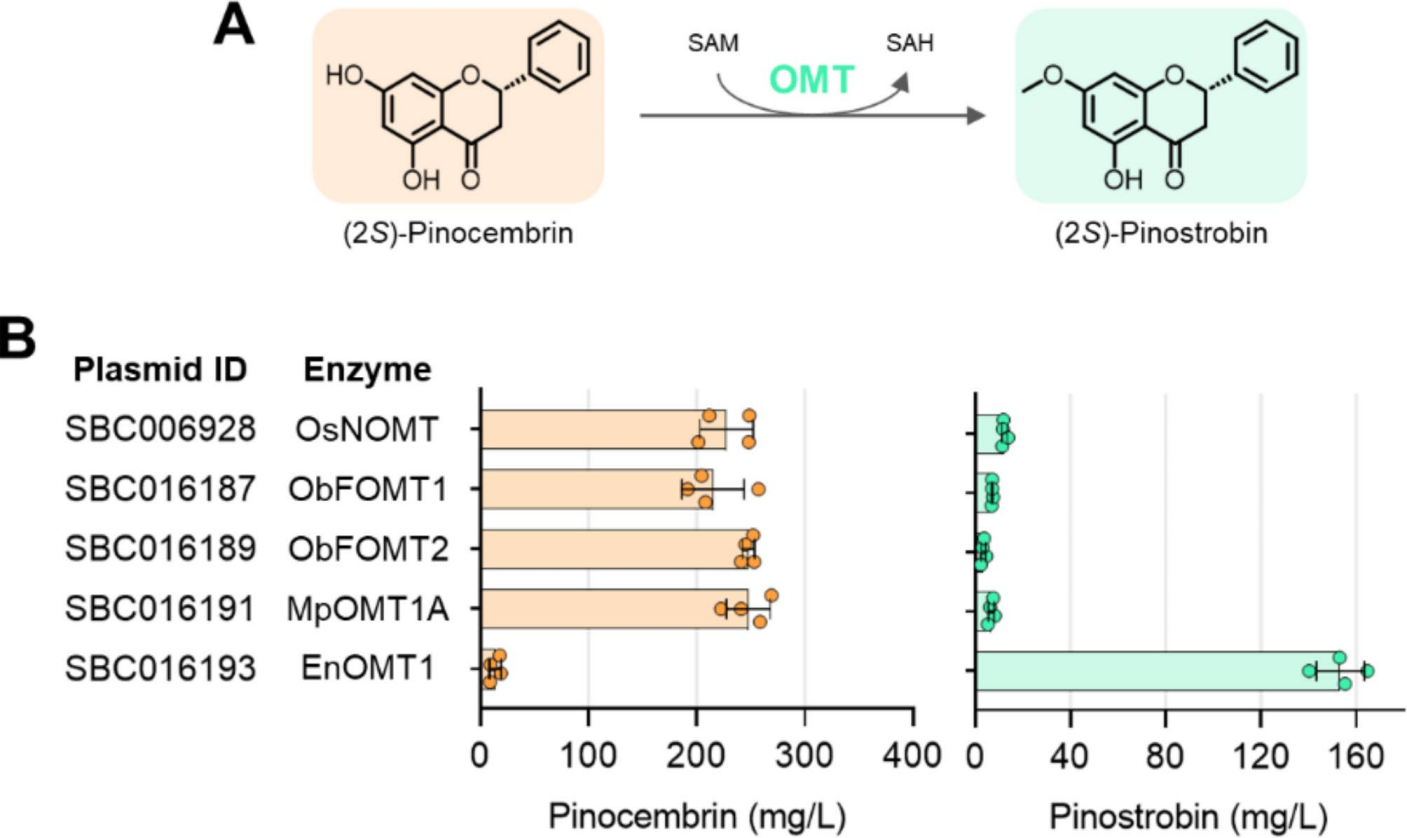
Biosynthesis of (2*S*)-pinostrobin using the *E. coli* pinocembrin chassis. **A** The *O*-methylation of (2*S*)-pinocembrin at the C-7 position is catalysed by flavonoid 7-*O*-methyltransferase (7-OMT). Cofactor abbreviations: SAM, *S*-adenosyl-l-methionine; SAH, S-adenosyl-l-homocysteine. **B** Production of pinostrobin in strain SBC016072 using a two-plasmid system. The first plasmid, SBC016090, encodes the optimised pinocembrin pathway, while the second plasmid encodes a library of different 7-OMT candidate enzymes. Pinocembrin (light orange) and pinostrobin (light green) titres are presented for the individual strains. Organism abbreviations: Os, *Oryza sativa* ssp. *japonica*; Ob, *Ocimum basilicum*; Mp, *Mentha piperita*; En, *Eucalyptus nitida*. Gene expression was induced by addition of IPTG. Data are presented as mean ± standard deviation, *n* = 4. Source data are available in the Source Data file

Pinostrobin, an *O*-methylated flavanone with antioxidant, anti-inflammatory, and anticancer properties [8, 36, 37], is primarily obtained through extraction from leaves of pigeon pea (*Cajanus cajan*) and rhizomes of fingerroot (*Boesenbergia rotunda*) [38, 39]. The biosynthesis of (*S*)-pinostrobin involves the *S*-adenosyl-l-methionine (SAM)-dependent *O*-methylation of (*S*)-pinocembrin at the C-7 position (Fig. 4A). To achieve this conversion, we examined a panel of five flavonoid 7-*O*-methyltransferases (7-OMT). All five candidate enzymes produced pinostrobin (Fig. 4B). However, while pinostrobin titres for OsNOMT, ObFOMT1, ObFOMT2, and MpOMT1A ranged from only 3 to 12 mg/L, the 7-OMT from *Eucalyptus nitida* (Smithton peppermint) produced up to 153 ± 10 mg/L pinostrobin, leaving only 14 mg/L pinocembrin unconverted. Unlike the four less effective 7-OMT candidates [40–42], the 7-OMT from *E. nitida* has previously been shown to exhibit the highest specificity toward pinocembrin as a flavonoid substrate [43], a finding confirmed in our in vivo screen.

### Production of pinobanksin and galangin

**Fig. 5:**
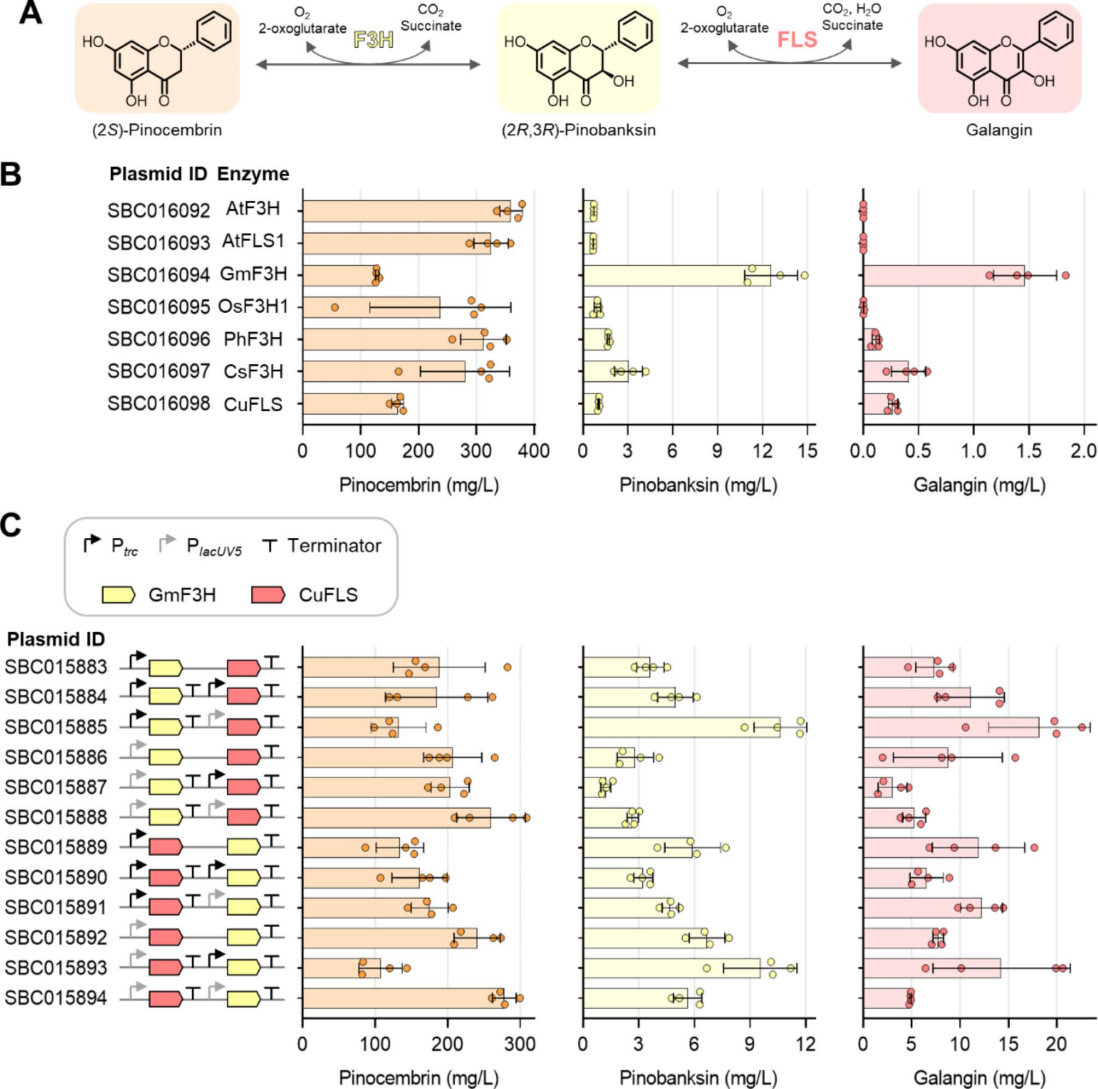
Biosynthesis of (2*R*,3*R*)-pinobanksin and galangin using the *E. coli* pinocembrin chassis. **A** The two-step conversion of (2*S*)-pinocembrin into galangin. The first step is catalysed by flavanone 3-hydroxylase (F3H), which forms the intermediate flavanonol pinobanksin. The second step is catalysed by flavonol synthase (FLS), forming galangin. **B** Production of pinobanksin and galangin in strain SBC016072 using a two-plasmid system. The first plasmid, SBC010507, encodes the optimised pinocembrin pathway, while the second plasmid encodes a library of different F3H and bifunctional FLS/F3H candidate enzymes. Organism abbreviations: At, *Arabidopsis thaliana*; Gm, *Glycine max*; Os, *Oryza sativa* ssp. *japonica*; Ph, *Petunia hybrida*; Cs, *Citrus sinensis*; Cu, *Citrus unshiu*. **C** Production of pinobanksin and galangin in strain SBC016072 carrying the optimised pinocembrin pathway, SBC010507, in conjunction with a combinatorial library of plasmids encoding GmF3H and CuFLS. Pinocembrin (light orange), pinobanksin (light yellow), and galangin (light red) titres are presented for the individual strains. Expression of genes was induced by addition of IPTG. Data are presented as mean ± standard deviation, *n* = 4. Source data are available in the Source Data file

The flavonol galangin can be synthesised from pinocembrin in two catalytic steps via the intermediate flavanonol (2*R*,3*R*)-pinobanksin. Initially, flavanone-3-hydroxylase (F3H) catalyses the hydroxylation of (2*S*)-pinocembrin at the C-3 position (Fig. 5A). Subsequently, flavonol synthase (FLS) introduces the double bond between the C-2 and C-3 positions, in a reaction similar to that catalysed by FNSI. In addition to their antioxidant properties [9], both pinobanksin and galangin have been reported to inhibit xanthine oxidase, making them potential candidates for the prevention and treatment of hyperuricemia or gout [44]. Previous attempts to produce galangin in both *E. coli* and *Streptomyces venezuelae* utilised *Citrus sinensis* (sweet orange) F3H and *Citrus unshiu* (satsuma mandarin) FLS [15, 45]. However, despite supplementing the cultures of recombinant *E. coli* with phenylalanine and cultures of recombinant *S. venezuelae* with pinocembrin, only low titres of galangin (1.1 mg/L and 1.0 mg/L, respectively) were achieved.

To optimise galangin production, we initially screened a library of seven F3H candidate enzymes for their ability to convert pinocembrin into pinobanksin. Considering that some FLS enzymes are capable of catalysing both reactions [46], we also included *C. unshui* FLS in the library screen and quantified galangin titres. All seven candidate enzymes successfully produced pinobanksin, with the F3H from *G. max* yielding the highest titre of 12.6 ± 1.8 mg/L (Fig. 5B). This particular enzyme has previously been shown to be the best candidate of a smaller enzyme library for the conversion of the flavanone naringenin into the flavanonol dihydrokaempferol [34]. Moreover, galangin production was observed for F3H enzymes from *G. max*, *O. sativa* ssp. *japonica* (japonica rice), *Petunia hybrida* (garden petunia), *C. sinensis*, and FLS from *C. unshiu*, suggesting their bifunctionality as F3H/FLS enzymes. While *G. max* F3H achieved the highest galangin titre of 1.5 mg/L, *C. unshiu* FLS exhibited the highest galangin to pinobanksin ratio (Supplementary Table S1), underscoring its primary function in catalysing the second reaction.

Since the F3H from *G. max* produced the most pinobanksin in our screen, and the FLS from *C. unshiu* yielded the best ratio of galangin to pinobanksin, we reasoned that combining the two genes for these enzymes into a single plasmid construct would further enhance our galangin titres. Therefore, we constructed a combinatorial library of plasmids encoding *G. max* F3H and *C. unshiu* FLS. Gene expression levels were varied through promoter strength and gene order (Fig. 5C). This library, previously utilised to produce the flavonol kaempferol from naringenin [34], yielded galangin titres ranging from 3.0 to 18.2 mg/L, showing up to a fivefold difference between the various combinations tested. The highest galangin titres were obtained in strains expressing F3H from a *trc* promoter and FLS from a *lacUV5* promoter (plasmids SBC015885 and SBC015893). The same strains also showed the highest titres of the intermediate pinobanksin, reaching a highest titre of 10.7 mg/L in the strain carrying plasmid SBC015885.

## Conclusion

In this study, we successfully constructed an *E. coli* chassis for pinocembrin production (353 ± 19 mg/L), using glycerol as the carbon source. Subsequently, we extended the heterologous pathway to enable the biosynthesis of the flavonoids chrysin (82 ± 5 mg/L), pinostrobin (153 ± 10 mg/L), pinobanksin (12.6 ± 1.8 mg/L), and galangin (18.2 ± 5.3 mg/L), also from glycerol. Despite achieving higher titres of these pinocembrin derivatives compared to previous studies in *E. coli* and *S. cerevisiae*, significant amounts of the intermediates cinnamate and pinocembrin remained unconverted. This suggests that further enhancements in production might be achieved by enzyme engineering, implementing additional strain modifications aimed at increasing the availability of malonyl-CoA to boost pinocembrin levels, and facilitating the supply and regeneration of cofactors, such as heme for chrysin production via FNSII enzymes. Overall, this study provides a foundation for further structural modifications of the microbially synthesised pinocembrin derivatives chrysin, pinostrobin, pinobanksin, and galangin, offering a more scalable approach for producing these pharmacologically valuable compounds.

## Materials and methods

### Enzyme selection

Enzyme candidates were selected based on both manual literature searches and Selenzyme output [47]. Sequences were scored based on the following criteria: known protein activity, reaction carried out, sequence similarity, and phylogeny. High-scoring candidates were compared with literature for evidence of functional expression in *E. coli* and affinity towards relevant substrates. Enzyme candidates are listed in Supplementary Table S2.

### Strains and media

*E. coli* NEB5α (New England Biolabs) was employed for plasmid propagation and cloning. *E. coli* MG1655 served as the base strain for constructing the pinocembrin chassis strain SBC016072, which was subsequently employed for the biosynthesis of chrysin, pinostrobin, pinobankin, and galangin. All strains used and generated in this study are listed in Supplementary Table S3. Bacterial cells were routinely cultivated in Luria-Bertani (LB) medium [48]. The production of flavonoids was performed in phosphate-buffered Terrific Broth (TBP, Formedium), supplemented with 0.4% (w/v) glycerol, and necessary antibiotics at the following concentrations: 100 µg/mL ampicillin, 20 µg/mL chloramphenicol, and 50 µg/mL kanamycin.

### Cloning and transformation

Plasmid DNA extraction was carried out using the QIAprep Spin Miniprep Kit (Qiagen). Oligonucleotide primers were synthesised by Integrated DNA Technologies (IDT) and are listed in Supplementary Table S4. DNA for cloning purposes was amplified by PCR using the Q5 High-Fidelity 2X Master Mix from NEB. Gel-purified linearised DNA fragments were extracted using the Zymoclean Gel DNA Recovery Kit (Zymo Research). Restriction enzymes, T4 DNA ligase, and the NEBuilder HiFi DNA Assembly Master Mix were purchased from NEB. Screening of bacterial colonies to confirm the chromosomal deletion or integration of genes was performed by colony PCR using the Phire Green Hot Start II PCR Master Mix (2X, Thermo Fisher) in 20 µL reactions. All PCR, digestion, ligation, and HiFi assembly reactions were set up following the manufacturer’s instructions. Chemical competent *E. coli* were prepared and transformed by heat shock [48].

### Plasmid construction

The gene parts were designed using PartsGenie [49], with genes optimised for *E. coli* codon usage and RBS translation initiation rates set to 20,000. Subsequently, they were synthesised and cloned into a pBbA1k-based expression vector by TWIST Bioscience [50]. The sequences of these gene parts can be found in Supplementary Table S5. Plasmids were constructed either through restriction enzyme-based cloning [48] or HiFi DNA assembly. A detailed description of how each plasmid was built is provided in the Supplementary Methods. All constructs were verified by Sanger sequencing (Eurofins Genomics). All plasmids used and generated in this study are listed in Supplementary Table S6.

### Strain construction

The individual gene deletion and genomic integration strains were constructed using CRISPR-Cas12a based genome editing as reported previously [51]. For the construction of the target-specific vectors used to delete *adhE*, *fabF*, *hcaE*, and *pta-ackA*, the target genes were screened for Cas12a protospacer adjacent motifs (PAMs) with the sequence TTTV. Subsequently, candidate PAM and protospacer sequences, each 27 bp long, were ranked using the CRISPR AsCpf1 insertion and deletion score web tool [52]. The protospacer sequence with the highest score was selected for vector construction. The donor DNA, comprising 50-bp homologous arms, was designed to delete all but the start codon and the final seven codons at the 3′ end of the target gene [51].

For the construction of the target-specific vector used to integrate CgACC (plasmid SBC016066), the *yjiP*_*yjiR* intergenic region, which has been shown to be a stable integration locus in *E. coli* [53], was screened for PAMs, and protospacer sequences were ranked similarly to those for the gene deletion vectors. In the case of this integration vector, the donor DNA comprised the genes encoding the three subunits of CgACC – *accBC*, *accD1*, and *accE* – optimised for *E. coli* codon usage and controlled by the *trc* promoter. This construct was flanked by 500-bp homologous arms. The sequences of the individual gene parts can be found in Supplementary Table S5. Similarly, the l-phenylalanine overproduction strain, SBC010792, was constructed by integrating a 8695-bp construct (encoding the *lacI* gene, and the *pheA*(G309C), *ppsA*, *aroF*(P148L), and *tktA* genes controlled by the *lacUV5* promoter) into the *lacZ* locus of the *E. coli* MG1655 strain. This construct was PCR-amplified from plasmid SBC008376 [54] and cloned in between the 500-bp *lacZ* homologous arms of plasmid SBC012918 (pTF-lacZ-rfp [51]) by HiFi DNA assembly, to create the target-specific integration vector pTF-lacZ-8376 (plasmid SBC016130).

Gene deletions and genomic integrations were confirmed by colony PCR using the primers listed in Supplementary Table S4. Selected strains were further verified by whole genome sequencing on a PacBio Sequel instrument, carried out in house or by PlasmidSaurus Inc. (Oregon, USA). Genomic DNA samples for colony PCR and whole-genome sequencing were prepared using a Monarch Genomic DNA Purification Kit (NEB).

### Strain cultivation and sample preparation

For the biosynthesis of flavonoids, single colonies of freshly transformed cells were used to inoculate 1 mL of TBP medium supplemented with 0.4% (w/v) glycerol and the relevant antibiotics. Cells were grown in 96-well deep well plates (DWP) sealed with breathable seals. Seed cultures were incubated at 30℃ and 80% humidity with orbital shaking at 850 rpm for 18 h. The main cultures were set up by diluting the seed cultures to an OD_600_ of 0.05 in 1 mL of fresh TPB medium supplemented with 0.4% (w/v) glycerol and the appropriate antibiotics in 96-well DWP. Optionally, for chrysin biosynthesis using FNSII enzymes, the medium was further supplemented with 0.1 mM 5-aminolevulinic acid. These main cultures were returned to the shaker-incubator. Upon reaching an OD_600_ of 1.0–2.0, isopropyl β-d-1-thiogalactopyranoside (IPTG) was added to a final concentration of 0.1 mM. The cultures were then returned to the shaker-incubator, and samples were collected after 24 h.

Samples for target compound quantification were prepared by transferring an aliquot of the bacterial culture to a 96-well microtitre plate and diluting it fourfold with 100% methanol. Subsequently, these samples were stored at − 80℃. On the day of analysis, the samples were thawed at room temperature, centrifuged for 10 min at 1,600 × *g*, and the supernatants were further diluted with 40% methanol to ensure they were within the linear range of quantification.

### Quantification of target compounds

For the quantification of cinnamic acid and pinocembrin, as part of the *E. coli* chassis strain development, UPLC-DAD analysis was used. For the quantification of pinocembrin and its derivatives, including chrysin, pinostrobin, pinobanksin, and galangin, LC-MS/MS analysis was employed.

UPLC-DAD analysis was performed using a 1290 Infinity II Agilent LC system equipped with a Waters ACQUITY BEH C18 column (50 mm × 2.1 mm × 1.7 μm) and a diode array detector measuring absorbance at 275 and 290 nm. The column temperature was maintained at 45℃. The separation was achieved using a flow rate of 0.5 mL/min and a binary mobile phase consisting of A (H_2_O, 0.1% formic acid, FA) and B (methanol, 0.1% FA). The gradient elution program was 0–1.5 min, 40–95% B; 1.5–1.9 min, held at 95% B; 1.9–2.0 min, 95–40% B; 2.0–2.5 min, held at 40% B. Samples were kept at 10℃ during the analysis and the injection volume was 5 µL. Peak areas were integrated using Agilent OpenLab software.

LC-MS/MS analysis was performed using a Waters ACQUITY UPLC H-Class Binary System coupled to a Waters Xevo TQ-XS Triple Quadrupole Mass Spectrometer. The chromatography method mirrored the UPLC-DAD method described above, except acetonitrile was used as solvent B instead of methanol, and the injection volume was 1 µL. MS parameters and MRM transitions for pinocembrin, chrysin, pinostrobin, pinobanksin, and galangin are provided in Supplementary Table S7. Peak areas were integrated using Waters MassLynx software. Metabolite concentrations were determined using calibration curves generated from standards of known concentrations.

## Electronic supplementary material

Below is the link to the electronic supplementary material.

Supplementary Material 1

Supplementary Material 2

## Data Availability

No datasets were generated or analysed during the current study.
