# Supplementary material for: Engineering an Escherichia coli strain for enhanced production of flavonoids derived from pinocembrin
Source: Microb Cell Fact. 2024 Nov 19;23:312. doi: 10.1186/s12934-024-02582-z (PMC11575205; doi:10.1186/s12934-024-02582-z)
Supplement: Supplementary file 2 — Supplementary Material 2 [file 12934_2024_2582_MOESM2_ESM.docx]

**Supplementary Information**

**Engineering an *Escherichia coli* strain for enhanced production of flavonoids derived from pinocembrin**

Erik K. R. Hanko, Christopher J. Robinson, Sahara Bhanot, Adrian J. Jervis, Nigel S. Scrutton*

Manchester Institute of Biotechnology, Faculty of Science and Engineering, University of Manchester, 131 Princess Street, Manchester M1 7DN, United Kingdom

*Author to whom correspondence should be addressed: Email: nigel.scrutton@manchester.ac.uk

# Supplementary Methods

## Plasmid construction

SBC016022 was constructed by HiFi DNA Assembly of two parts. The first part contains the *adhE* spacer and left homology arm and was amplified by PCR from pTF-tyrR using primers EHfbrh_001/EHfbrh_002. The second part contains the right homology arm and vector backbone and was amplified by PCR from pTF-tyrR using primers EHfbrh_003/EHfbrh_004.

pTF-fabF was constructed by HiFi DNA Assembly of two parts. The first part contains the vector backbone and was amplified by PCR from pTargetF using primers pTFopen-F/pTFopen-R. The second part contains the *fabF* spacer and the left and right homology arms and was amplified by PCR from a linear DNA construct synthesised by Twist Bioscience using primers pTF-fabF_F/pTF-fabF_R.

SBC016023 was constructed by HiFi DNA Assembly of two parts. The first part contains the *pta* spacer and left homology arm and was amplified by PCR from pTF-tyrR using primers EHfbrh_005/EHfbrh_006. The second part contains the right homology arm and vector backbone and was amplified by PCR from pTF-tyrR using primers EHfbrh_007/EHfbrh_004.

SBC016066 was constructed by HiFi DNA Assembly of seven parts. The first part contains the pTF vector backbone and was amplified by PCR from pTF-tyrR using primers EHfbrh_044/EHfbrh_004. The second part contains the spacer sequence and was amplified by PCR from pTF-tyrR using primers EHfbrh_032/EHfbrh_033. The third part contains the left homology arm and was amplified by PCR from *Escherichia coli* MG1655 genomic DNA using primers EHfbrh_034/EHfbrh_053. The fourth part contains the *trc* promoter and Cg*accBC* and was amplified by PCR from gene part CgAccBC using primers EHfbrh_054/EHfbrh_037. The fifth part contains Cg*accD1* and was amplified by PCR from gene part CgAccD1 using primers EHfbrh_038/EHfbrh_039. The sixth part contains Cg*accE* and was amplified by PCR from gene part CgAccE using primers EHfbrh_040/EHfbrh_041. The seventh part contains the right homology arm and was amplified by PCR from *Escherichia coli* MG1655 genomic DNA using primers EHfbrh_042/EHfbrh_043.

SBC016090 was constructed by restriction enzyme-based cloning. The chloramphenicol resistance marker from pBbE5c-rfp was cloned into SBC010507 using AatII/SpeI restriction sites.

SBC016091 was constructed by restriction enzyme-based cloning. The ampicillin resistance marker from pBbE5a-rfp was cloned into SBC010507 using AatII/SpeI restriction sites.

SBC016092 was constructed by restriction enzyme-based cloning. The gene AtF3H (Q9S818), including ribosome binding site, was cloned from SBC014335 into pBbA1c-rfp using EcoRI/BamHI restriction sites.

SBC016093 was constructed by restriction enzyme-based cloning. The gene AtFLS1 (Q96330), including ribosome binding site, was cloned from SBC014337 into pBbA1c-rfp using EcoRI/BamHI restriction sites.

SBC016094 was constructed by restriction enzyme-based cloning. The gene GmF3H (Q53B69), including ribosome binding site, was cloned from SBC015610 into pBbA1c-rfp using EcoRI/BamHI restriction sites.

SBC016095 was constructed by restriction enzyme-based cloning. The gene OsF3H1 (Q7XM21), including ribosome binding site, was cloned from SBC015612 into pBbA1c-rfp using EcoRI/BamHI restriction sites.

SBC016096 was constructed by restriction enzyme-based cloning. The gene PhF3H (Q07353), including ribosome binding site, was cloned from SBC015614 into pBbA1c-rfp using EcoRI/BamHI restriction sites.

SBC016097 was constructed by restriction enzyme-based cloning. The gene CsF3H (Q9ZWR0), including ribosome binding site, was cloned from SBC015616 into pBbA1c-rfp using EcoRI/BamHI restriction sites.

SBC016098 was constructed by restriction enzyme-based cloning. The gene CuFLS (Q9ZWQ9), including ribosome binding site, was cloned from SBC015618 into pBbA1c-rfp using EcoRI/BamHI restriction sites.

SBC016130 was constructed by by HiFi DNA Assembly of two parts. The first part comprises the linearised pTF-lacZ-rfp vector for cargo DNA insertion and was amplified by PCR from pTF-lacZ-rfp using primers pTF-lacZ-cargo-F/pTF-lacZ-cargo-R. The second part comprises the cargo DNA from SBC008376 and was amplified by PCR from SBC008376 using primers UP-F/PEPS-R.

# Supplementary Figures


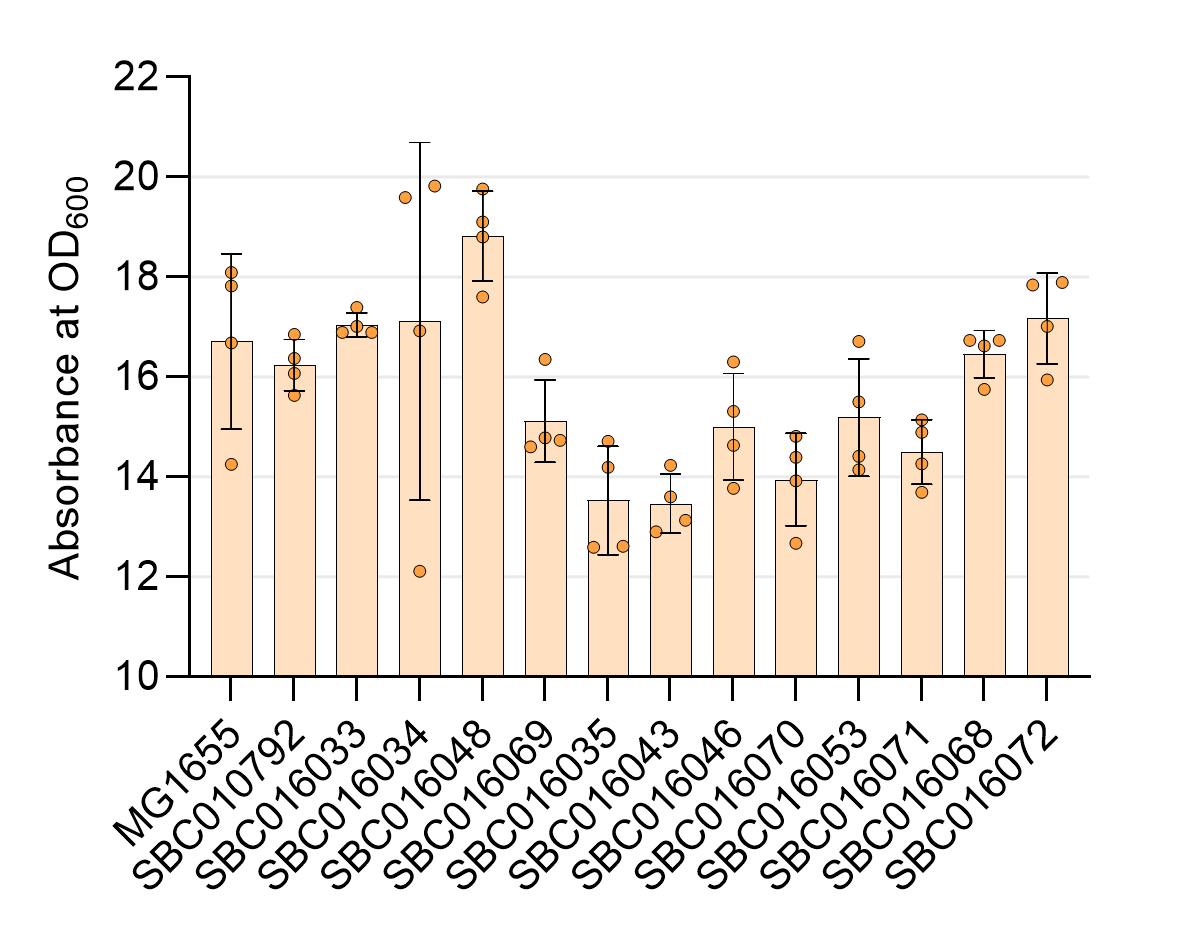


**Supplementary Figure S1.** Cell densities of strains carrying the pinocembrin biosynthesis pathway, plasmid SBC010507, 24 hours after cultures had been supplemented with the pathway inducer IPTG. Data are presented as mean ± standard deviation, n = 4. Source data are available in the Source Data file.


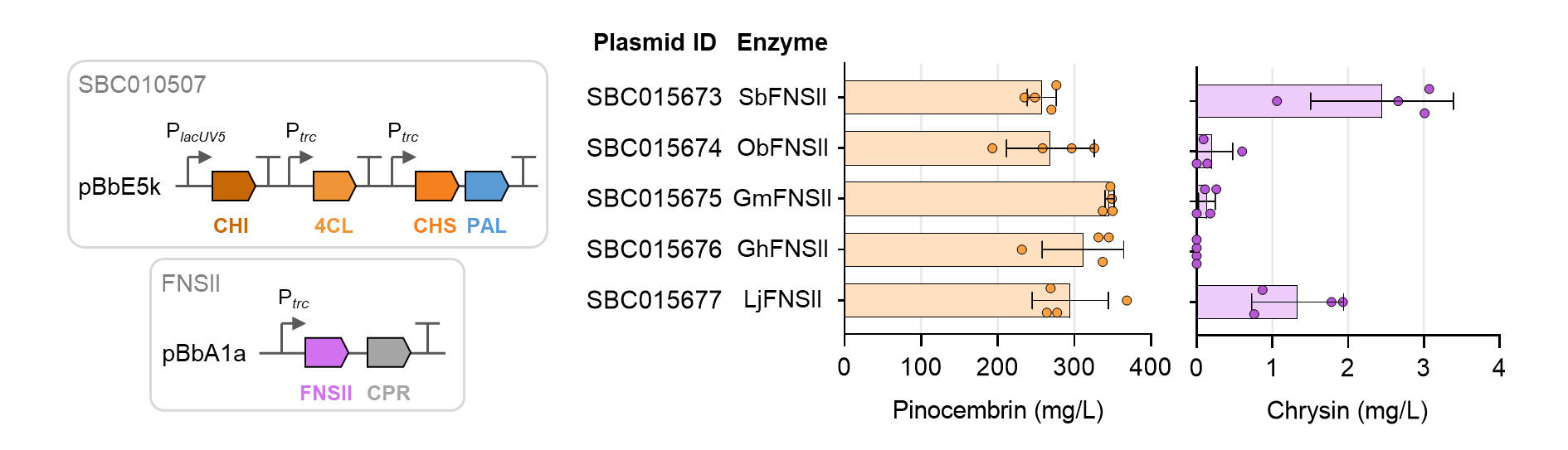


**Supplementary Figure S2.** Biosynthesis of chrysin in strain SBC016072 using a two-plasmid system. Plasmid SBC010507 encodes the optimised pinocembrin pathway, while the second plasmid encodes a library of different FNSII candidate enzymes in conjunction with Arabidopsis thaliana CPR. Pinocembrin (light orange) and chrysin (light purple) titres are presented for the individual strains. Organism abbreviations: Sb, Scutellaria baicalensis; Ob, Ocimum basilicum; Gm, Glycine max; Gh, Gerbera hybrida; Lj, Lonicera japonica. Strains were grown in the absence of 5-aminolevulinic acid. Expression of genes was induced by addition of IPTG. Data are presented as mean ± standard deviation, n = 4. Source data are available in the Source Data file.

# Supplementary Tables

**Supplementary Table S1.** Galangin to pinobanksin ratio of strain SBC016072 carrying the pinocembrin biosynthesis pathway (SBC010507) in conjunction with a library of different F3H/FLS candidate enzymes. Titres are given as mean, n=4. Source data are available in the Source Data File.

| **Plasmid ID** | **Enzyme** | **Pinobanksin (nM)** | **Galangin (nM)** | **Ratio galangin:pinobanksin (nM/nM)** |
| --- | --- | --- | --- | --- |
| SBC016092 | AtF3H | 2654 | 0 | 0 |
| SBC016093 | AtFLS1 | 2490 | 0 | 0 |
| SBC016094 | GmF3H | 46193 | 5403 | 0.117 |
| SBC016095 | OsF3H1 | 3527 | 5 | 0.001 |
| SBC016096 | PhF3H | 6217 | 413 | 0.066 |
| SBC016097 | CsF3H | 11154 | 1505 | 0.135 |
| SBC016098 | CuFLS | 3827 | 1000 | 0.261 |

**Supplementary Table S2.** Enzymes used in this study.

| **Enzyme name** | **Uniprot (UniParc) ID** | **Species source** | **Reference** |
| --- | --- | --- | --- |
| OsNOMT | Q0IP69 | *Oryza sativa* subsp. *japonica* | *^1^* |
| ObFOMT1 | K0I977 | *Ocimum basilicum* | *^2^* |
| ObFOMT2 | K0II72 | *Ocimum basilicum* | *^2^* |
| MpOMT1A | Q6VMW2 | *Mentha piperita* | *^3^* |
| EnOMT1 | UPI0020684696 | *Eucalyptus nitida* | *^4^* |
| AtF3H | Q9S818 | *Arabidopsis thaliana* | *^5^* |
| AtFLS1 | Q96330 | *Arabidopsis thaliana* | *^6^* |
| PcFNSI | Q7XZQ8 | *Petroselinum crispum* | *^7^* |
| PaFNSI | A0A076U8J8 | *Plagiochasma appendiculatum* | *^8^* |
| CcFNSI | A6XFT1 | *Cuminum cyminum* | *^9^* |
| AcFNSI | A6XFT2 | *Aethusa cynapium* | *^9^* |
| AgFNSI | Q4QUR8 | *Apium graveolens* | *^10^* |
| DcFNSI | Q4QUR9 | *Daucus carota* | *^10^* |
| AaFNSI | A6XFT4 | *Angelica archangelica* | *^9^* |
| CmFNSI | B3RFV8 | *Conium maculatum* | *^10^* |

**Supplementary Table S3.** Strains used and constructed in this study.

| **Strain name or ID** | **Characteristic** | **Reference or source** |
| --- | --- | --- |
| *Escherichia coli* NEB5α | *fhuA2Δ(argF-lacZ)U169 phoA glnV44 Φ80Δ(lacZ)M15 gyrA96 recA1 relA1 endA1 thi-1 hsdR17* | NEB |
| *Escherichia coli* MG1655 | Wild type | DSMZ |
| SBC010792 | *Escherichia coli* MG1655 *lacZ*::*lacI*-P*_lacUV5_*-Ec*pheA*(G309C)-Ec*ppsA*-Ec*aroF*(P148L)-Ec*tktA*-T_7_ | This work |
| SBC016033 | SBC010792 Δ*adhE* | This work |
| SBC016034 | SBC010792 Δ*pta-ackA* | This work |
| SBC016035 | SBC010792 Δ*fabF* | This work |
| SBC016043 | SBC010792 Δ*fabF* Δ*adhE* | This work |
| SBC016046 | SBC010792 Δ*fabF* Δ*pta-ackA* | This work |
| SBC016048 | SBC010792 Δ*adhE* Δ*pta-ackA* | This work |
| SBC016053 | SBC010792 Δ*fabF* Δ*pta-ackA* Δ*adhE* | This work |
| SBC016068 | SBC010792 Δ*fabF* Δ*pta-ackA* Δ*adhE* *yjiP*-*yjiR*::P*_trc_*-Cg*accBC*-Cg*accD1*-Cg*accE*-T*_rrnB2_* | This work |
| SBC016069 | SBC010792 Δ*adhE* Δ*pta-ackA* *yjiP*-*yjiR*::P*_trc_*-Cg*accBC*-Cg*accD1*-Cg*accE*-T*_rrnB2_* | This work |
| SBC016070 | SBC010792 Δ*fabF* Δ*hcaE* | This work |
| SBC016071 | SBC010792 Δ*fabF* Δ*pta-ackA* Δ*adhE* Δ*hcaE* | This work |
| SBC016072 | SBC010792 Δ*fabF* Δ*pta-ackA* Δ*adhE* Δ*hcaE* *yjiP*-*yjiR*::P*_trc_*-Cg*accBC*-Cg*accD1*-Cg*accE*-T*_rrnB2_* | This work |

**Supplementary Table S4.** Oligonucleotide primers used in this study.

| **Primer name** | **Sequence (5′ to 3′)** | **Purpose** |
| --- | --- | --- |
| EHfbrh_001 | gtaatttctactcttgtagatgaagacgcggtagaaaaagcagatttttttgaagcttgggccc | Plasmid assembly |
| EHfbrh_002 | cgctttcataatgctctcctgataatgttaaacttttttagtaaatcatctgctcgcgtaccctaggtataaacgcag |  |
| EHfbrh_003 | gagagcattatgaaagcgaaaaaatccgcttaatcagtagcgctgtctggcaacataaacggtgaattctctagagtcgacctg |  |
| EHfbrh_004 | atctacaagagtagaaattactagtattatacctaggac |  |
| EHfbrh_005 | gtaatttctactcttgtagatcccagtctctgaactacgaaatctttttttgaagcttgggccc |  |
| EHfbrh_006 | gcagactgcatggaagtacctataattgatacgtggctaaaaaaacgtcagggagccacgtaccctaggtataaacgcag |  |
| EHfbrh_007 | cttccatgcagtctgcacagcagcagtaatctcgtcatcatccgcagctttgcgctgctgaattctctagagtcgacctg |  |
| EHfbrh_032 | agtaatttctactcttgtagatatgggttatgtagacctgataagtttttttgaagcttgggccc |  |
| EHfbrh_033 | cttccagcacaaacgcgtaccctaggtataaacgcag |  |
| EHfbrh_034 | cgtttgtgctggaaggtcac |  |
| EHfbrh_037 | tcggtacagggcaattatttgatttctagtagcactacccctt |  |
| EHfbrh_038 | ttgccctgtaccgatgcaa |  |
| EHfbrh_039 | ggttttgatttggttacagaggcatgttgccgt |  |
| EHfbrh_040 | ctgtaaccaaatcaaaacctccactgagc |  |
| EHfbrh_041 | ggccttctctcgagtttggatccgagtctacaatcaaaagaagttaacgttctgaaaggc |  |
| EHfbrh_042 | ctcgagagaaggccatcctgacggatggcctttttagcgcatcaggctgctt |  |
| EHfbrh_043 | gtcgactctagagaattcagttcccgctggtggatgt |  |
| EHfbrh_044 | tgaattctctagagtcgacctgc |  |
| EHfbrh_053 | cacaattccacacattatacgagccggatgattaattgtcaaacctggtcaggcgttcac |  |
| EHfbrh_054 | gtataatgtgtggaattgtgagcggataacaatttcagaattcaaaagatcttttaagaaggagatatacatatgtccgtggaaacccgt |  |
| pTF-lacZ-cargo-F | catatggaattcctgcagtgc |  |
| pTF-lacZ-cargo-R | ctcgaatggtctatatcctacg |  |
| UP-F | caggaattccatatgcttcccaaccttaccagaggg |  |
| PEPS-R | tatagaccattcgaggagatccttactcgagtttggatcc |  |
| pTFopen-F | tgaattctctagagtcgacctgc |  |
| pTFopen-R | atctacaagagtagaaattactagtattatacctaggac |  |
| pTF-fabF_F | aggtataatactagtaatttctactcttgtagatgcgc |  |
| pTF-fabF_R | actctagagaattcacccctcgttacg |  |
| EHseqF002 | aatcacagtgagtgtgagcg | Colony PCR for deletion of *adhE* |
| EHseqF003 | ggaagccgttatagtgcctc |  |
| fabF_chk-Fw | cagcttacatcacgggtgaaactttgc | Colony PCR for deletion of *fabF* |
| fabF_chk-Rv | cgaatcaatacttgctcaagacgattg |  |
| EHseq049 | gcgacgaaatagcgtaaatgc | Colony PCR for deletion of *hcaE* |
| EHseq050 | cgtgtttgcgcgttaactg |  |
| EHseqF004 | gccattggctgaaaattacgc | Colony PCR for deletion of *pta-ackA* |
| EHseqF005 | gcaattcattgatgcagcgc |  |
| lacZflank-F | gcttgctgcaactctctcag | Colony PCR for genome insertion at *lacZ* locus |
| lacZflank-R | gatggtttgcccggataaacg |  |
| EHseqF015 | gccatcagcagctttaatgtg | Colony PCR for genome insertion at *yjiP*-*yjiR* locus |
| EHseqF016 | acggtagccgtagtccttac |  |

**Supplementary Table S5.** Sequences of the synthesised gene parts. Gene coding sequences are shown in uppercase letters. EcoRI and BamHI restriction enzyme recognition sites are italicised.

| **Gene** | **Sequence** |
| --- | --- |
| AaFNSI | *gaattc*aaaagatctgagtcttgtagacgtaatattgatgcctagacagcacgaggagccgggtccttataacctgcctttatccggacaaatatattcaaggaaggaaggttgaaATGGCTCCGACAACGATTACCGCCTTAGCTCAGGAGAAAACCCTGAATTTAGCATTTGTCCGCGACGAAGATGAACGTCCTAAAGTCGCGTATAACCAATTTTCGAACGAAATCCCGATTATTAGCTTGGCGGGAATGGATGATGATACAGGGCGCCGCCCGCAAATTTGTCGTAAAATTGTTGAAGCTTTTGAAGATTGGGGTATATTTCAGGTGGTGGATCATGGCATCGACGGGACCTTAATTTCCGAGATGACCCGGTTAAGCCGTGAGTTCTTCGCGTTACCGGCCGAGGAGAAACTCCGCTACGATACGACGGGCGGCAAACGTGGCGGCTTTACCATCTCGACGCATCTTCAGGGTGATGATGTGAAAGATTGGCGTGAATTTGTCACGTACTTTTCGTACCCAATCGATGATCGCGACTATTCGCGTTGGCCCGATAAACCGCAGGGATGGCGTAGTACAACCGAAGTGTATAGCGAAAAGCTGATGGTGCTGGGCGCGAAACTGTTGGAAGTGCTAAGCGAGGCTATGGGTCTAGAGAAAGAAGCGCTGACTAAGGCGTGTGTAAATATGGAACAGAAGGTGTTAATCAATTACTACCCCACCTGCCCCGAACCCGACCTAACACTGGGCGTGCGCCGTCATACAGATCCAGGTACCATTACGATCCTGCTGCAGGACATGGTGGGAGGATTGCAAGCTACACGCGATGGAGGTAAAACTTGGATTACGGTCCAGCCTGTAGAGGGCGCGTTTGTTGTTAACCTGGGCGATCACGGACATTATCTCTCAAACGGCCGATTTAAGAACGCGGATCACCAAGCAGTCGTTAATAGCACCAGTTCACGGTTGTCTATTGCCACCTTTCAAAACCCCGCCCAAAACGCGATCGTATACCCGTTGCGCATCCGTGAAGGCGAAAAAGCCGTCCTGGATGAAGCGATTACGTACGCGGAAATGTACAAAAAGAATATGACCAAGCACATAGAAGTCGCTACCCTGAAAAAGTTAGCGAAGGAAAAACGTCTGCAAGAAGAAAAGGCGAAACTGGAAACTGAAAGTAAAAGCGCCGATGGCATTAGCGCGTAAttgtagactc*ggatcc* |
| AcFNSI | *gaattc*aaaagatctgagtcttgtagatatctggcctatacacagttgctgcatggtgtctaaaagcaataggtataaaatatttgaccgtttatacacgctggaggaggttattcATGGCACCGACTACCATCACCGCACTGTCACAGGAAAAATCGCTTAATCTGGACTTTGTCCGTGATGAAGATGAACGTCCTAAAGTAGCGTATAATCAATTCAGCAATGAAATCCCGATTATCTCATTAGCCGGCATGGATGATGATTCGAACGGCCGTCGCCCGGAGATTTGCCGTAAGATTGTGGAAGCATTTGAAGACTGGGGCATTTTCCAGGTGGTGGACCACGGCATTGACAAGGGCCTGATCTCTCAAATGTCGCGGCTCAGTCGCGAGTTCTTTGCGCTGCCTGCAGAAGAAAAGCTCCGTTACGACACGACCGGAGGAAAACGAGGTGGCTTCACCATCAGCACTCACCTACAGGGCGATGACGTGAAAGATTGGCGTGAATTTGTAACGTATTTCTCGTATCCGATTGAAGACCGCGATTACAGCCGTTGGCCAGAAAAACCAGAGGGTTGGCGGTCGACCACCGAAGTTTATTCAGAGAAACTTATGGTATTGGGTGCCAAGTTGTTAGAAGTGCTGTCCGAGGCGATGGGCTTGGAAAAGGAAGCCCTGACGAAAGCATGCGTGAATATGGAGCAAAAGGTTCTCATAAATTATTATCCGACCTGTCCGGAACCGGATTTGACCCTTGGCGTCCGTCGTCACACGGACCCCGGAACGATTACGATTCTGTTGCAGGACATGGTGGGCGGCCTGCAGGCAACCCGTGATGGCGGCAAAACCTGGATTACGGTGCAACCGGTTGAGGGCGCATTTGTGGTCAACCTGGGCGACCACGGTCATTATCTTAGCAACGGTCGTTTCAAAAATGCCGACCATCAGGCGGTGGTTAATAGCACCTCTAGCCGTCTCTCTATTGCGACTTTTCAGAATCCCGCCCAGAATGCAATCGTGTATCCGTTGAAAATCCGCGAGGGTGAAAAGGCGATTCTGGACGAAGCTATCACCTATGCGGAAATGTATAAGAAAAATATGACCAAACATATCGAAGTTGCTGCCCTGAAGAAATTGGCGAAGGAAAAACGCCTGCAGGACGAAAAAGCCAAACTGGAAATGTAAttgtagactc*ggatcc* |
| AgFNSI | *gaattc*aaaagatctgagtcttgtatggaacccaaagtgatcgaggtcgaaaacactagttatttggaactatagtgtaccgcacaagttcgacactaaaggaagttcaaATGGCTCCGTCCACCATTACCGCATTATCTCAGGAGAAAACTTTAAACTTAGACTTTGTTAGGGATGAAGACGAACGTCCTAAAGTTGCGTATAACCAGTTCTCAAATGAGGTTCCGATCATATCCTTAGCCGGCCTTGACGATGATAGCAACGGCCGCCGTGCAGAAATTTGCCGCAAAATTGTTGAGGCTTTCGAAGAATGGGGCATATTTCAGGTGGTGGATCACGGCATCGATTCGGGTCTGATTAGCGAAATGAGCCGTTTGTCCCGTGAATTTTTCGCGCTGCCGGCGGAAGAAAAACTTGTTTACGATACCACCGGGGAGAAAAAGGGCGGTTTCACCATCAGCACCCATTTGCAGGGTGATGACGTTCGCGATTGGCGTGAATTTGTGACATACTTTTCATACCCTATTTCAGCGCGTGATTACTCACGTTGGCCCAAAAAGCCAGAAGGCTGGCGTAGTACCACCGAAGTGTATAGCGAAAAGCTGATGGTGCTGGGCGCAAAGCTGTTGGAAGTGTTGTCGGAGGCAATGGGTTTAGAAAAAGAAGCGTTAACTAAAGCATGTGTCGAAATGGAGCAGAAAGTGCTGATTAACTACTACCCTACGTGCCCAGAACCGGACCTGACGCTGGGCGTACGCCGCCATACCGATCCGGGGACCATCACCATTTTGCTTCAAGATATGGTCGGTGGATTGCAGGCGACGCGTGATGGAGGCAAAACGTGGATCACAGTTCAGCCGGTAGAAGGTGCGTTTGTCGTGAATTTAGGCGATCACGGTCATTATCTTTCTAACGGCCGCTTTCGTAATGCGGATCATCAGGCAGTGGTAAATAGTACATCTACACGCCTTTCGATTGCCACCTTTCAGAACCCGGCCCAGAACGCGATTGTCTATCCTTTAAAAATTCGGGAGGGCGAAAAGGCCATTCTGGATGAAGCCATCACATACGCTGAAATGTACAAAAAGAACATGACCAAACACATTGCAGTGGCTACACAGAAGAAACTGGCTAAGGAAAAGCGCCTGCAAGATGAAAAGGCTAAGATGAAAATCTGAttgtagactc*ggatcc* |
| AtF3H | *gaattc*aaaagatctgagtcttgtatgaccgcaagatagtccccgtttctaaacaagtgggccggcgccccggccggcagaagttgggaataaggaggttggatacgcagATGGCACCGGGGACGCTTACGGAATTAGCGGGGGAATCGAAACTGAACTCCAAATTTGTGCGCGATGAGGATGAGAGGCCAAAAGTAGCGTATAATGTGTTTAGCGATGAAATCCCGGTGATTTCACTGGCCGGAATCGATGATGTGGACGGGAAACGCGGCGAAATCTGCCGCCAGATTGTCGAAGCGTGCGAGAACTGGGGCATATTTCAGGTGGTAGATCACGGCGTGGATACCAACCTGGTCGCGGATATGACACGCCTCGCCCGCGATTTCTTCGCGCTGCCACCCGAGGACAAACTGCGTTTTGATATGTCAGGCGGTAAGAAAGGGGGTTTCATAGTGAGCTCTCACCTGCAAGGCGAGGCGGTGCAGGATTGGCGCGAAATCGTCACTTATTTCAGCTACCCAGTTCGTAACAGGGATTATAGCCGCTGGCCTGATAAACCGGAGGGGTGGGTTAAAGTTACCGAAGAGTACAGCGAACGCCTTATGTCGCTGGCATGTAAGCTGCTGGAAGTGCTGTCCGAAGCCATGGGACTTGAGAAGGAAAGTCTGACCAACGCGTGCGTGGATATGGATCAAAAGATCGTAGTGAACTACTACCCGAAATGTCCCCAACCCGATCTGACGCTCGGCTTAAAACGTCACACGGACCCCGGTACCATCACGCTACTGTTGCAGGATCAAGTGGGCGGCTTACAGGCAACTCGTGACAATGGTAAAACGTGGATCACTGTCCAACCGGTTGAAGGTGCATTTGTGGTGAACTTGGGCGATCACGGCCATTTCCTGTCCAACGGCCGTTTTAAAAATGCTGATCACCAGGCGGTGGTCAATAGCAATTCCTCGCGCCTGAGCATTGCCACCTTTCAGAACCCGGCGCCGGACGCGACCGTCTACCCGCTGAAAGTTCGTGAAGGTGAGAAAGCGATTCTGGAAGAACCGATCACATTTGCGGAAATGTACAAACGTAAGATGGGCCGTGATTTGGAATTGGCCCGCCTCAAAAAGCTGGCCAAAGAAGAACGCGATCATAAAGAAGTGGATAAACCTGTGGATCAAATTTTTGCGTGAttgtagactc*ggatcc* |
| AtFLS1 | *gaattc*aaaagatctgagtcttgtacagatgcatgtggctttgtaatcatagcagttctagcctggtgctttaggcagcagggggattattcagaagataggtgaggagaaatgatATGGAGGTCGAACGCGTGCAAGACATCAGTAGCAGCTCGCTGCTAACGGAAGCCATCCCGCTTGAATTTATCCGCAGCGAAAAAGAACAGCCGGCGATCACCACCTTTCGTGGTCCTACGCCGGCCATCCCGGTCGTCGACTTAAGCGATCCGGACGAAGAATCAGTGCGCCGCGCAGTTGTAAAAGCAAGCGAGGAATGGGGCCTCTTTCAAGTTGTGAATCATGGTATTCCGACAGAACTGATACGGAGACTGCAGGATGTGGGTCGTAAATTCTTCGAATTGCCGAGTTCGGAAAAGGAATCCGTGGCGAAACCCGAGGATTCTAAAGACATTGAAGGCTATGGCACAAAACTACAGAAAGACCCGGAGGGCAAGAAGGCCTGGGTTGATCATCTGTTCCACCGTATTTGGCCGCCGTCCTGCGTGAACTACAGGTTTTGGCCGAAGAATCCTCCGGAATATCGTGAAGTAAACGAAGAGTACGCCGTACACGTTAAAAAGTTATCGGAAACCCTGCTGGGAATCCTAAGTGATGGCTTAGGCCTGAAACGTGACGCACTGAAGGAAGGCCTGGGAGGAGAAATGGCGGAGTACATGATGAAAATCAATTACTACCCGCCGTGTCCGCGCCCGGATTTAGCGCTGGGAGTGCCCGCGCATACCGATCTGAGTGGCATTACGTTACTGGTACCGAACGAGGTCCCTGGCCTGCAGGTGTTCAAAGATGATCACTGGTTTGATGCAGAATACATTCCTTCGGCCGTGATCGTACATATTGGTGACCAGATTTTACGCTTGAGCAATGGGCGCTACAAGAATGTCCTGCATCGTACGACTGTGGATAAAGAGAAAACCCGTATGAGCTGGCCGGTATTTCTGGAGCCGCCGCGGGAGAAGATCGTTGGTCCTTTGCCGGAGCTTACGGGAGATGATAACCCGCCGAAGTTTAAACCGTTTGCATTTAAAGATTACTCCTATCGTAAACTGAATAAACTGCCTCTGGATTAAttgtagactc*ggatcc* |
| CcFNSI | *gaattc*aaaagatctgagtcttgtaatgaatctcaatcgtatccgattttctcagactagaggacacctgttgtaaacccgcctgaggacgtgtttgacgtataagaaagggggtctacaacgATGGCGCCGACCACGATTACTGCTCTGGCTCAGGAGAAAACCCTTAATTCGGATTTTGTTAGGGACGAGGACGAGCGCCCGAAGGTGGCCTATAATCAATTTAGCACTGAGATTCCGATCATTAGCCTCGCGGGGATCGACGACGACAGTAAAGGTCGCCGTCCTGAGGTGTGTCGCAAAATCGTTGAAGCGTTCGAAGACTGGGGCATTTTTCAGGTTGTAGATCATGGCGTAGATTCAGCATTAATTTCCGAGATGTCACGTTTAAGTCGCGAGTTCTTTGCGCTGCCAGCCGAGGAAAAGTTACGCTATGACACGACTGGGGGCAAGCGTGGAGGCTTCACGATAAGTACCCACCAGCAAGGCGACGATGTGCGTGACTGGCGCGAGTTCGTGACGTACTTTTCCTACCCCGTCGATGCCCGTGATTACTCCCGTTGGCCGGAAAAGCCGGAAGGTTGGCGGTCTGTAACCGAGGTTTACAGTGAAAAGTTAATGGTTCTTGGCGCTAAACTGCTGGAGGTATTGTCGGAGGCCATGGGCTTGGACAAAGGCGCACTCACCAAAGCTTGCGTTAATATGGAACAGAAAGTCCTGATCAATTACTATCCTACCTGTCCTGAACCGGACCTGACCCTGGGTGTTCGCCGTCATACCGATCCCGGGACTATTACGATTTTACTCCAGGATATGGTTGGCGGTCTGCAAGCCACGCGTGATGGTGGCAAAACCTGGATCACCGTACAACCGGTTGAGGGTGTCTTCGTCGTGAATCTCGGAGATCATGGGCATTATCTATCAAATGGGCGTTTCAAAAACGCCGACCATCAGGCAGTGGTGAACAGCACCTCCAGCCGGTTATCTATTGCCACGTTTCAGAATCCCGCCCAGAACGCCATTGTATATCCCCTCAAAATTCGCGAAGGCGAAAAGCCGATTCTGGAAGAGGCGATTACCTACGCCGAAATGTACAAGAAAAATATGACCAAACACATCGAAGTAGCAACGCAGAAGAAACTGGCCAAAGAAAAACGCCTGCAGGAAGAGAAAGCCAAGCTGGAAACCAAAACAAAATCTGCAGACGGTATCTTGGCATAAttgtagactc*ggatcc* |
| Cg*accBC* | *gaattc*aaaagatctgagtcttgtaatgagcttgctctggtgaacttttgcttgccagggctgcacaaactgcagtgcaattaaattattttcaaaacatagaggtcaaagaATGTCCGTGGAAACCCGTAAAATTACAAAAGTGCTGGTGGCGAATCGCGGAGAAATTGCTATTCGGGTATTTCGGGCGGCGCGCGATGAAGGCATTGGGTCGGTGGCAGTGTATGCCGAACCAGATGCCGACGCACCCTTTGTTAGCTACGCCGATGAGGCGTTTGCTCTAGGAGGACAAACCTCGGCAGAAAGCTATCTAGTTATTGATAAAATAATAGATGCGGCTCGTAAGTCGGGTGCAGATGCCATCCATCCTGGCTACGGCTTCCTGGCGGAAAACGCTGATTTTGCCGAGGCCGTGATCAACGAGGGGCTCATTTGGATCGGTCCGTCCCCGGAAAGCATTCGTTCCCTGGGGGACAAAGTGACCGCCAGACACATCGCGGATACAGCGAAAGCCCCCATGGCCCCGGGCACGAAAGAACCGGTTAAAGACGCCGCCGAAGTGGTAGCCTTTGCCGAGGAATTTGGCTTACCAATAGCAATTAAAGCGGCATTTGGCGGTGGCGGTCGAGGCATGAAGGTAGCCTATAAAATGGAAGAAGTGGCGGATCTCTTCGAAAGTGCGACGCGTGAGGCTACTGCGGCGTTTGGACGGGGTGAGTGCTTCGTAGAACGCTACCTGGATAAAGCTCGTCACGTGGAAGCACAAGTCATTGCCGATAAACATGGCAACGTCGTTGTTGCAGGTACGCGCGACTGCTCGTTACAGCGCCGCTTCCAGAAACTGGTTGAAGAAGCCCCGGCGCCTTTTCTGACCGATGACCAGCGCGAGCGTCTGCACAGCAGTGCCAAAGCAATTTGTAAAGAAGCGGGTTATTATGGTGCCGGTACCGTGGAGTATTTGGTGGGTAGCGATGGCCTGATATCATTTCTGGAAGTCAACACGCGCCTTCAGGTAGAGCACCCGGTGACCGAAGAGACGACCGGTATTGATTTGGTTAGGGAAATGTTTCGCATTGCAGAAGGTCATGAACTGAGCATTAAAGAAGATCCGGCCCCACGCGGCCATGCTTTTGAATTTCGAATCAACGGGGAAGATGCTGGTAGTAACTTTATGCCGGCACCGGGCAAAATTACTAGTTATCGTGAACCCCAGGGACCTGGGGTGCGGATGGACAGCGGTGTCGTGGAGGGCAGCGAAATTAGCGGCCAGTTTGATTCAATGTTGGCTAAACTAATCGTTTGGGGAGATACTCGCGAACAGGCTTTGCAGCGTTCACGCCGTGCTTTGGCGGAGTACGTAGTTGAAGGTATGCCTACCGTTATTCCGTTTCATCAACATATCGTGGAAAATCCTGCTTTTGTGGGCAACGACGAAGGATTTGAGATTTATACAAAGTGGATCGAAGAAGTATGGGACAACCCGATTGCGCCTTACGTGGATGCGTCGGAACTGGATGAGGATGAAGACAAGACCCCAGCACAGAAAGTGGTCGTTGAGATTAACGGCCGACGTGTTGAAGTGGCGCTGCCGGGCGATTTGGCATTGGGCGGTACCGCGGGTCCGAAAAAGAAAGCGAAGAAACGTAGGGCGGGCGGAGCAAAGGCGGGTGTGAGCGGTGACGCAGTCGCGGCGCCGATGCAGGGTACCGTGATCAAAGTTAACGTTGAGGAGGGCGCAGAGGTAAATGAAGGCGACACTGTGGTGGTGCTGGAGGCCATGAAAATGGAAAACCCCGTCAAAGCACATAAAAGTGGCACAGTAACTGGACTGACGGTAGCTGCCGGGGAAGGCGTCAATAAAGGGGTAGTGCTACTAGAAATCAAATAAttgtagactc*ggatcc* |
| Cg*accD1* | *gaattc*aaaagatctgagtcttgtattgccctgtaccgatgcaacctgttttgtgccgaaacattgcgctaatcaagcgtacagggtttcacaaaaagaggaggtcaaATGACTATTAGTAGTCCGTTAATTGACGTGGCCAATCTGCCGGATATTAACACGACGGCAGGCAAAATTGCCGATCTAAAAGCTCGTCGTGCAGAGGCGCACTTTCCGATGGGCGAAAAAGCTGTGGAAAAAGTCCATGCCGCCGGCCGTTTAACCGCGCGCGAACGTTTGGACTACCTTCTGGACGAAGGAAGCTTCATCGAAACCGACCAGCTGGCGCGTCATCGCACGACCGCTTTCGGCTTAGGCGCGAAGCGTCCTGCGACTGACGGTATTGTGACCGGCTGGGGCACGATCGACGGCCGCGAGGTGTGCATTTTCAGTCAGGATGGCACTGTTTTCGGTGGTGCGCTCGGGGAAGTGTATGGCGAAAAGATGATTAAGATCATGGAACTCGCCATTGATACGGGACGTCCGCTAATTGGTCTGTATGAGGGCGCGGGCGCCCGTATCCAGGACGGAGCTGTGAGCCTGGACTTTATTAGTCAGACGTTTTATCAGAACATCCAGGCAAGCGGCGTCATTCCGCAGATCAGTGTGATTATGGGCGCTTGCGCTGGTGGGAATGCGTACGGTCCGGCCTTGACCGATTTCGTGGTGATGGTTGATAAAACGAGTAAAATGTTCGTGACGGGACCAGACGTGATCAAAACCGTTACCGGGGAAGAGATTACACAGGAGGAACTGGGCGGAGCCACCACGCATATGGTGACGGCAGGCAACTCGCACTATACAGCGGCAACTGATGAAGAAGCCTTAGATTGGGTGCAGGATCTTGTGTCTTTTCTTCCGTCAAACAACCGTTCTTACGCGCCGATGGAAGATTTTGACGAGGAAGAGGGCGGTGTCGAAGAAAATATCACTGCTGATGACTTAAAACTGGACGAAATCATTCCAGATAGCGCTACCGTCCCGTACGATGTGCGCGATGTGATCGAATGCCTGACGGATGATGGGGAGTACCTGGAAATTCAAGCCGATCGTGCCGAAAATGTCGTGATTGCGTTTGGCCGCATCGAAGGACAGTCCGTTGGTTTTGTGGCGAACCAGCCAACGCAGTTTGCCGGGTGCCTCGATATCGATTCGAGCGAAAAAGCGGCCCGTTTCGTGCGTACCTGCGACGCCTTCAACATCCCCATAGTGATGTTGGTCGATGTGCCGGGTTTTCTACCAGGAGCAGGACAGGAATATGGTGGTATCCTGCGCCGAGGTGCCAAACTGCTGTATGCATACGGCGAGGCGACCGTGCCAAAAATTACCGTTACGATGCGTAAAGCCTATGGTGGAGCGTATTGTGTCATGGGATCAAAGGGTCTGGGGTCGGATATTAACCTCGCATGGCCGACGGCCCAGATTGCCGTAATGGGCGCGGCGGGGGCGGTAGGCTTCATTTACCGCAAAGAGCTTATGGCCGCGGATGCCAAAGGCCTGGATACTGTGGCGCTGGCCAAAAGTTTTGAACGCGAATATGAAGATCACATGCTTAATCCTTACCATGCCGCCGAACGTGGTCTGATTGATGCGGTGATTCTCCCTTCAGAAACCCGAGGCCAGATTAGTCGTAACCTTCGTTTATTAAAACATAAAAACGTGACGCGTCCGGCCCGCAAGCACGGCAACATGCCTCTGTAAttgtagactc*ggatcc* |
| Cg*accE* | *gaattc*aaaagatctgagtcttgtaccaaatcaaaacctccactgagcctttccgcaggaatgtatgtttctgccgcttgtgcctgctccatccaggcgtatataggagataacATGAGTGAAGAAACTACCCAAGATACGAAGGCCGCGGAAAAGCCGTTCCTCCAAATTGTCAGCGGGAACCCGACGGATCAGGAAGTCGCCGCGTTGACGGTGGTGTTCGCGGGACTGGCGAAAGCGGCGGCGGCGCAGCAGATGGTATCGGCATCAAAAGATAGGAATAATTGGGGAAACTTAGATGAACGTTTATCTCGCCCGAATACCTTTAATCCTAGCGCCTTTCAGAACGTTAACTTCTTTTGAttgtagactc*ggatcc* |
| CmFNSI | *gaattc*aaaagatctgagtcttgtactcaactggtcgcacaacacgcatgagctcgcgccgttcggcggatcgtgtaattcgaatatagacgacgaagatagaggttcATGGCGCCAACCACCATCACTGCACTAGCACAGGAAAAGACCTTGAACCTGGCCTTCGTACGTGATGAAGATGAACGCCCTAAAGTTGCGTACAATGAATTTAGCAATGAAATTCCAATTATCTCGCTTGCCGGTTTAGAAAACGATAGTGATGGCCGTCGTCCAGAAATTTGTCGTAAAATTGTGGAGGCTTTCGAAAACTGGGGCATTTTCCAAGTTGTCGATCACGGCATTGACAGCGCACTGATCTCGGAAATGTCGCGGCTGAGCCGCGAATTTTTCGCCTTGCCCGCCGAAGAAAAACTGCGGTATGATACAACCGGCGGTAAACGTGGTGGTTTTACAATTAGCACGCACTTACAAGGCGATGATGTGCGCGATTGGCGCGAATTTGTGACGTATTTTTCATACCCTATTGACGCCCGCGATTACAGTCGTTGGCCTGATAAACCTGAAGGCTGGCGCTCAATAACCGAAGTTTATTCGGAACGCCTGATGGTATTGGGCGCCAAGTTGCTTGAAGTTTTGTCGGAGGCAATGGGCCTAGAAAAAGAAGCCTTGACCAAGGCCTGTGTCAACATGGAGCAGAAAGTGTTAATCAATTACTACCCGACATGTCCGGAACCAGACCTGACATTAGGGGTGCGACGTCATACCGATCCAGGTACGATCACCGTGCTGCTGCAAGATATGGTTGGTGGCCTCCAGGCCACTCGTGACGGCGGCAAAACATGGATTACCGTCCAGCCGGTGGAAGGTGCTTTTGTGGTTAATCTGGGTGACCACGGCCACTACTTATCTAACGGTCGCTTTAAGAACGCCGACCACCAGGCAGTGGTAAATAGTAGCAGCAGCCGGCTGTCTATTGCCACATTTCAAAACCCTGCGCAGAACGCCATCGTGTACCCGTTAAAGATTCGTGAGGGGGAGAAGGCTATTTTAGATGAAGCCATTACCTACGCCGAGATGTACAAAAAGAACATGACAAAACACATCGAAGTGGCGACACTGAAGAAACTCGCGAAAGAGAAACGTCTGCAGGATGAGAAAGCGAATATGGAAAAGAAGTCGAAATCAGCGCACGGCATCTCGGCTTAAttgtagactc*ggatcc* |
| DcFNSI | *gaattc*aaaagatctgagtcttgtactgccaatcactttcgccggcgcatgtgcgcgtaagcgacaccgcaaagttcctcaactcgacaaaagggggttccgcataATGGCACCAACAACTATCACGGCCCTGGCAAAAGAAAAGACACTGAACAGCGACTTCGTTCGTGATGAAGATGAACGCCCGAAAGTGGCCTATAATCAGTTCTCGACCGAAATTCCCATTATCAGCCTGGCGGGTATTGACGATGATTCCAACGGCCGTCGCCCGGAAGTCTGCCGAAAGATTGTGGAGGCGTTTGAGGACTGGGGTATTTTTCAGGTAGTGGATCACGGCATTGATTCAGGCTTAATTGCCGAAATGTCCCGCCTTTCACGCGAATTTTTCGCCCTGCCGGCAGAAGAGAAATTGCGCTATGACACGACCGGTGGAAAACGAGGCGGATTCACGATTAGCACCCACCTGCAGGGAGACGATGTAAAAGATTGGCGCGAATTTGTAGTGTACTTTAGTTATCCGGTAGACGCACGCGATTACTCACGCTGCCCTGATAAACCGGAGGGCTGGCGTTCGGTGACCGAAGTTTATTCTGAGAAATTGATGGCCCTGGGAGCCAAATTGCTCGAAGTACTTAGCGAAGCGATGGGCTTGGAAAAAGAAGCCCTGACGGAAGCCTGTGTGAATATGGAGCAAAAAGTTTTGATTAATTATTACCCGACCTGTCCGCAACCGGATTTAACCCTGGGTGTGCGCCGCCACACCGACCCGGGAACCATAACCATTCTGCTTCAGGACATGGTTGGCGGTCTGCAGGCCACCCGTGACGGTGGCAAAACCTGGATTACGGTTCAACCCGTTGAGGGAGCCTTCGTGGTCAACCTGGGAGATCACGGCCACTACCTCTCGAACGGCCGATTCAAAAATGCAGACCATCAGGCCGTTGTTAACTCCACGTCGAGCCGTCTTAGCATCGCTACCTTTCAGAACCCGGCTCAGAACGCAATTGTGTATCCGTTGAAAATTCGCGAGGGAGAAAAACCTATTCTGGAAGAGGCAATGACATACGCAGAAATGTACAAGAAAAACATGACGAAGCACATTGAAGTTGCCACTCAGAAGAAACTCGCGAAAGAAAAGCGTCTCCAGAACGAAAAAGCCAAACTGGAAACCAAATTTTGAttgtagactc*ggatcc* |
| EnOMT1 | *gaattc*aaaagatctgagtcttgtacgtaaggcaatgtatcgcgattcaggctttagaacagaatatgttcatatctacaattgcatttatactggatagggaggcacgatcATGGACCCGCTGGATGAAACGTTACGTGGCCAAGCCCAAGCCATGAAACACATGTATGCCTTTGCCGATTCAATGGCTCTTAAATGTGCCGTAGAATTACGTATCCCTGACATTATCCATTCAGAAGGAGCCGGTCCTGTTACCCTGGCCCAAATCGCGAGCCGCATCCCTTCGCCGAGCCCAGAAATGGCATATCTGGCCTCGATTATGCGCCTGCTGGTGTGCAAGAACGTGTTTTCTGCAGTGAGGAACGGCGGTGAAACCTTGTATGGCTTAACTCCTACTAGCCGTTGGTTACTGCAAGGGGGCGGCCATCTGTCACTTGCGCCCGCTGTGCTATTAGTTTCGCACCCAGCGATGATGTCACCGTGGCATAATCTGAGCGACTGCATCAAAGATGGCGGCTCAACGGCTTTCCAGATGGCGCACGGTCGCGGTCTTTGGGATTTAGCGACAGAGAACCCAGAATTTAATCGTACCTTTCGCGAAGGCATGGCCTGCCTGAACAAACTTATGATGAAAGCCATTGTCGATGCCTACAAGGATGGCTTTGAGCGAGTTGGTTCAATGGTGGATGTGGGTGGGGGCACCGGCGATGCCGTAAGCGAAATTGTTCGTCTTTATCCCCACATTAAAGGCATAAATTTCGACCGCCCGCACGTCATTGCGGAAGCACCAGCACACGGGGGCGTGTCGCACGTAGGTGGCGATATGTTTGAGGCGATCCCTTCTGCGGATGCGGTGTTTATGAAATGGGTCCTGCACAATTGGAATGATGAAGATTGCGTCCGTATTTTGAAAAATTGCAAGAAAGCGCTGCCGAAAAAGAATGGTGTAGTGATTATTGCAGATGTTGTGCTTCAGCCGGATGGCGATGGCCTGTTTGAAGAGGCGCAGGTTGGTCTGGACTTAATGATGATTACCGTAACGCGCGGCAAAGAGCGGAGTGAACCGGAATGGAAGAAAATTTTAGAAGAAGGTGGCTTTCCCCGCTACAATATTATCCAGACCAGCTGCATGTTAAGCATAATCGAAGCGTTTCCGTTATAAttgtagactc*ggatcc* |
| MpOMT1A | *gaattc*aaaagatctgagtcttgtacccggcttctgtttgggcccgtttggttcgttagctggaggcacctccgatttgttattacccggagaaggaggtggattATGGCCCCGGAGGAAGATTCTTTAGCACTGGCAGAAGCGTGGAACCATGGTTTCGGCTTTATTAAAACATCGATTGTGAAAACGGCGGTCGAACTGGAAATCCCTGATATTCTGGAGAGCCGTGGCGCCCCAGTGTCTATCCCAGAACTCGCCACAGCCGTGGATTGCTCAGCTGACCGCATTTACCGGGTAATGCGCTTCCTTGCCTATCATGGCATTTTCAAACGCACGAAACCTCCCCCGGAATCTACCGAGGGCGGCTCGGTGTATTATGCACAAACGCCCGTTTCGCGCCGGCTTACCCGTGAAAATCTGGGTCCTTTTGTTCTCCTGCAGGGTACTATGCGCGAACCATCGGGTTGCGTGACGGCTGAAACGCTACGGACCAGCAAACGCCCGGGCGTAGTCAACGAAAACGAAAGCGACCATTTATACGAAGATCCGGTGTTCAGCATGAAAGTGTTTCGAGATGCCATGGCGAGCCATGCCCGTATGACCACCGCGGCAGTGATCGAAAATTACGGGGAAGGCTTTCAGGGCGTGGGTTCGTTGGTCGACGTCGGCGGCAGCTATGGCATGGCGCTCAGTATGCTGGTGAAAGCATTCCCCTGGCTTCGCGGTATCTGCTTTGACCTCCCTGAAGTTGTTGCCCGCGCCTCACCGCTGAAGGGTGTGGAATTTGTCGGGGGCACCATGTTTGAATCCATACCGAAAGCGGATGTAGTTATGCTGATGTTCGTCCTCCATAACTGGTCAGACGAAGAATGTGTGGAGATTCTCAAGCGTTGCAAAGATGCGGTTTCGAAAGATAAAGGCAAAGTCATCATTATCGACGCGGTGATTGATGAGGATGGTGACGGCGACGAGTTTACGGGCGCGCGCCTTGGTTTAGATGTTACGATGATGGCGACCATGTTTGAGGGACGCGAACGCACCTACGTTGAATGGGCGCGTATAATCAACGAGGCTGGCTTTCGCCGTCATGTGGTGAAAAATATCAAAACTTTAGAAAGTGTAATTGAAGCATATCCCTGAttgtagactc*ggatcc* |
| ObFOMT1 | *gaattc*aaaagatctgagtcttgtactctactgtgttactgcactatttcgcaggtaacacatgaaattagaacaccgaatcccatatgggggaggagggaggtgatttaataacaATGGGTCGTGATGAAGAAGCGGCAGCGCAGGCGGAAGCGTGGAACCACGGCTTTGGTTTCATTAAAACGAGCGTAATTAAAACCGCCATTGAACTGGAAATCCCGGATATTTTGCACAACCAGGGCGGTCCTCTTTCGTTGAGCGCGCTGAGTAGCGCAGTGGGCGTACCTCCGGATCGTCTTCATCGTATCATGCGGTTCCTCGCCCACCATGGCGTGAGTAAGAAGACGGCATCTCCTCCTGGCGAATCAGACTATTATTATGCTGAGACTGCGGTTTCCCGCAGCCTCACTAAGGATAACCTGGGTCCATTTGTACTGTTACAGGGCGCGCAGCGTGGCCCTAGTGCATGCATCACTGCCCAAGGCTTAAAATCGCGTGAACGCCCGGGTGTGGAAGAACTGGGCAGTGACCCTCTCTACGAAGATCCGATCTTTACCGAGAAAGTGTTCCGCGATGCAATGACCTGCCATGCCAGGGTGACGACCTCTGCCGTTATTGAAAACTACGGCGAAGGCTTTCGTGGTGTCGGCAGCCTGGTGGACGTGGGCGGCTCCTACGGCATGACCCTGGGTATGCTGGTGGAGGCTTTCCCGTGGATTCGCGGCATTTGTTACGACCTGCCACCGGTCGTGGCGAAAGCAAAGCCCCTGCATGGAGTTGAATTTGTGGCAGGCTCGATGTTTGAAAGTGTGCCGAAAGCGGATGTTATCATGCTTATGTTCGTTCTGCACAATTGGAGCGATAATGAATGCATCGATATCCTTAAGCGCTGTAAAGAAGCCATACCTGCGGAAACCGGCCGTCTGATGATTATCGACGCAATTATTGATGAGGACGGTGAAGGCGATGAATTTGCTGGAGCCCGTCTGGGCCTGGACGTAACCATGATGGCCGTGACATATGAAGGCAAAGAACGTACGCACCGTGAGTGGGCATATATCCTGACCGAAGCGGGATTCCGTAAATATGTCGTCAATAATATCAAAGCCCTCGAAAGTCTTATCGAGGCCTACCCATAAttgtagactc*ggatcc* |
| ObFOMT2 | *gaattc*aaaagatctgagtcttgtatttgggtagtattctctaataggcttgtctgcatccgtgcccatgtctactggatagaactagactaatgagtatagatcagataaggagggataATGGGTCGAGATGAAGAAGCCGCCGCGCGCGCCGAAGCGTGGAACCATGGATTTGGCTTTATTAAAACCAGCGTAATTAAGACCGCGATTGAGCTTGAAATTCCCGATATTCTTCATAATCACGGCGCCCCTCTGAGCCTTAGCGCCCTGTCTTCTGCGGTTGGAGTTCCTCCGGATCGTCTGCACCGCATCATGCGCTTTCTGACCCACCATGGTGTTAGCAAGAAAACCGCCAGCCCGCCGGGCGAAAGCGATTATTACTACGCTGAAACGGCCGTTAGCCGCAGCCTGACCAAAGACAACCTGGGTGCCTTCGTTTTACTGCAGGGTGCGCAGCGTGGCCCATCGGCGTGCATTACGGCACAGGGCTTGAAATCGCGAGAGCGTCCGGGAGTAGAAGAACTGGGGAGCGACCCGTTATATGAAGACCCGATTTTTACAAAAATGGTGTTTCGCGATGCCATGGCATGCCATGCCCGCCTGACGACCTCAGCCGTCATTGAAAATTATGGCGAGGGCTTTCGCGGTGTAGGCAGTCTGGTTGATGTGGGTGGTAGTTACGGCATGACTTTAGGCATGCTGGTAGAAGCGTTCCCGTGGATTCGTGGCATTTGCTACGATTTACCGCAGGTTGTAGCTAAGGCGAAACCTTTACATGGAGTTGAGTTCGTTGCGGGTAGCATGTTTGAAAGCGTGCCGGAAGCTGACGTAGTGATGCTCATGTTCGTACTCCATAACTGGTCGGACAACGAATGCATTGACATTCTGAAACGTTGTAAAGAAGCGATCCCACGTGAAACTGGCAAAGTTATGATTATCGACGCGATTATAGAAGAAGATGGCGAAGGAGACGAGTTTGCGGAAGCGCGTTTAGGGCTAGATGTCACGATGATGGCAGTTACCTTCGAGGGCAAGGAACGCACCCACCGTGAATGGGCCTTCATCTTAAAAGAAGCAGGCTTCCGCAAGTATGTTGTGAAAAATATTAAAGCTCTGGAATCCCTGATTGAGGCGTACCCGTGAttgtagactc*ggatcc* |
| OsNOMT | *gaattc*aaaagatctgagtcttgtaacgttgaggaattacgcgcctgattttggaaatactgcatggatatggtattttgtgagaaaactcgctacgaaggaggttgtaaaATGGTTTCCCCAGTGGTCCACCGCCATGCCGCAGGCGGTGGTTCGGGCGGCGACGATGATGATCAGGCGTGTATGTATGCGCTGGAACTGCTCGGCGGCTCAGTCGTTTCGATGACGCTTAAAGCGGCCATTGAGCTCGGCCTGGTAGATGAACTGTTGGCAGCAGCGGGGGCGGCGGTTACCGCGGAAGAACTTGCAGCGCGTTTACGCCTCCCGGCCGCGGTGGCGGCGGCGGCGGCTGTGGATCGCATGTTACGTCTCCTGGCAAGTTATGGCGTGGTTCGCTGTGCGACCGAAGCTGGTCCCGACGGTAAAGCCCGCCGCTCGTACGCGGCGGCTCCGGTTTGCAAGTGGCTCGCGGCAGGCTCTTCGAGCGGAGAAGGTTCGATGGCGCCGCTTGGCCTTCTGAATCTGGATAAGGTATTCATGGAAAACTGGTACTATTTGAAGGAAGCGGTTTCTGAAGGTGGAACAGCATTCGATAAAGCCTACGGAACCTCGTTATTTCAGTACTTGGGTCAAGATGGAAACGAGCCATCTAATACCCTGTTCAACCAGGCAATGGCTTCACACTCCGTGGTCATTACTAATAAATTATTACAGTTTTTCCGGGGTTTTGATGCGGGCGCCGGTGTTGATGTTCTTGTAGATGTTGGGGGTGGCGTTGGTGCGACGCTGCGCATGATCACCGCGCGCCATCCGCACCTGCGTGGCGTTAATTATGACCTTCCGCACGTAATTGCACAAGCACCGCCTGTCGAGGGCGTAGAGCATATCGGCGGATCGATGTTTGATCACGTACCGTCGGGCTCTGCCATACTGTTGAAATGGATTCTGCACTTGTGGGGTGACGAGGAATGTGTGAAAATTCTGAAAAACTGCTACAAAGCCTTGCCGGCGAAGGGCAAGGTGATTCTTGTTGAGTACGTTCTGCCGGCAAGTCCTGAAGCGACCCTTGCGGCCCAAGAGGCATTTCGCCTGGATGTTATGATGCTGAACCGCCTGGCGGGTGGAAAAGAACGTACACAGCAGGAATTTACGGATCTCGCGGTAGATGCGGGCTTTTCTGGTGATTGCAAACCGACATACATCTTCACGAACGTATGGGCTCTGGAGTTCACGAAATAAttgtagactc*ggatcc* |
| PaFNSI | *gaattc*aaaagatctgagtcttgtaccgcgaggtgacactctcaacctgtccatcacgtcgatggagagaacagtttacacagtatgtgcaagaaggaggtacaaagATGGCTCCGCCAGCAGTAGCCGAATTTCCTAATGATACTAAACCGGTGGTAGCAGCGTCGGTGATGAAGCTGGCCAACGAGCTGACAGTTCTGCCTGAAAAATTTGTCAAAGCCGTGGGCGAGCGCCCTGTAACTGCGCACAATGACTATTGTAAAGAAATTCCGGTCATTTCTCTGAAAGGGATTGATAACGAATCGGAGCGTGCCCGCATCGTTGCCGAGGTGGGTCGCGCGTGTGCGGACTGGGGGATTTTCCAGATTGTCGATCATGGCATGTCCGCAGTGCTCATGAAACGGACCATGGAAACGGTCCTTGAGTTTTTCAAACTGCCCGTCGAAGAGAAACTGAAGTGCGCGGCGAAACCAGGCGACTTTCCGGTAGGATATGCGAGCGGATCGCACCGCGCCTCAGATGATGTGCTGGATTGGCGCGAATTTATGGCACATCGCTCATTGCCTAAAGCAATTCGCGAAAATGATATTTCGACCTGGCCGGATAAACCTGAGCATTATCGCAAAACCCTGGTTGAGTATTGTGATGCGTGCGATGGCCTGGTAACCGCCTTATTAGGCCTGATTTCGGAATCGCTGGGCCTGCCGACCAATTATATCAAGAACTTTGTTGGCGGCGATGATGCTGATCAAAAGATGATTCTGAATTATTACCCACAATGTCCCATGCCTGACCTGACATTAGGCTTACGCAGCCATACGGACTATGGCACCATTACGGTGCTCCAGCAAGACCAAGTTGGCGGTCTGCAAGTGTACAACGCCGATCGTCGTAAATGGGTGACAGTTGAACCCATTCCGGGTGCTTTAGTTGTGAACTTAGCGGATCAGCTGCAGATTTTAAGTAATAATAAATACTGCTCAGTGATGCACCAGGCCATTGTCAACAGTAATCACACCCGTCTGAGTATAGCGACACTTAGTAATCCGAACGCCATGTCCCAAATGGGACCGGCCCCGGAACTCCTCTCAGCGGAAAATCCGGCGAAATATCGTACGTACAAGTTCAAAGAGTACCTGCCGATTTGCATCGCCAAGAAAGCTACAAATCACTGTGATGCTGTAGCCCTATAAttgtagactc*ggatcc* |
| PcFNSI | *gaattc*aaaagatctgagtcttgtataaagcagtatcccgtatgaaatatagcgccatgaggtaaccacaagacctttggccaccacggtacgaatcttggctcaaaaggaggctccATGGCACCCACTACCATCACTGCACTGGCGAAAGAGAAGACCCTCAATTTAGATTTTGTACGCGATGAAGATGAACGTCCAAAAGTTGCATACAATCAGTTCTCCAATGAAATACCTATTATTTCTTTAGCCGGTCTTGATGATGATTCGGACGGCCGACGCCCGGAAATATGCCGCAAAATTGTCAAAGCTTGCGAAGACTGGGGAATCTTTCAGGTGGTAGATCACGGCATCGATAGCGGGCTTATTTCGGAAATGACCCGTTTATCACGTGAATTTTTCGCATTGCCAGCGGAAGAGAAATTGGAATACGATACCACCGGCGGCAAACGTGGCGGCTTCACCATTAGTACCGTTCTACAGGGGGATGACGCGATGGACTGGCGCGAGTTTGTCACGTACTTTTCGTACCCCATTAATGCCCGAGACTATTCACGCTGGCCGAAAAAGCCGGAAGGTTGGCGCAGCACAACCGAGGTTTATAGTGAAAAGCTGATGGTGCTGGGGGCGAAACTGCTTGAAGTTCTGAGTGAAGCGATGGGCTTGGAAAAAGGCGATCTTACGAAAGCGTGCGTGGATATGGAGCAGAAAGTGCTGATTAATTACTATCCAACCTGCCCGCAGCCCGACCTGACCCTCGGCGTTCGTCGTCACACCGATCCGGGCACCATCACCATTCTGCTGCAAGATATGGTTGGGGGCTTGCAAGCAACACGCGATGGCGGAAAAACTTGGATTACCGTTCAACCAGTAGAAGGCGCCTTTGTGGTTAATCTGGGTGATCACGGCCATTACTTATCGAATGGTCGTTTTCGTAATGCAGACCACCAAGCCGTGGTTAATTCGACGAGCTCTCGCCTGAGCATCGCGACCTTCCAGAACCCGGCCCAGAATGCCATTGTGTATCCTCTTAAAATTCGAGAAGGAGAGAAAGCCATTTTAGACGAAGCCATTACGTATGCCGAAATGTACAAGAAATGCATGACCAAACATATTGAGGTGGCCACCCGTAAGAAGCTGGCAAAAGAAAAACGTCTCCAAGATGAAAAAGCGAAACTCGAAATGAAAAGCAAATCCGCAGATGAGAACCTTGCGTAAttgtagactc*ggatcc* |

**Supplementary Table S6.** Plasmids used and generated in this study.

| **Plasmid ID** | **Characteristic** | **Reference or source** |
| --- | --- | --- |
| pBbA1c-rfp | Chl^R^, p15A, P*_trc_*-rfp-T*_rrnB1_* | *^11^* |
| pBbE5a-rfp | Amp^R^, ColE1, P*_lacUV5_*-rfp-T*_rrnB1_* | *^11^* |
| pBbE5c-rfp | Chl^R^, ColE1, P*_lacUV5_*-rfp-T*_rrnB1_* | *^11^* |
| SBC016022 | pTF vector for deletion of *adhE* | This work |
| pTargetF | Spec^R^, gRNA scaffold | *^12^* |
| pTF-fabF | pTF vector for deletion of *fabF* | This work |
| pTF-hcaE | pTF vector for deletion of *hcaE* | *^13^* |
| SBC016023 | pTF vector for deletion of *pta-ackA* | This work |
| pTF-tyrR | pTF vector for deletion of *tyrR* | *^14^* |
| SBC006928 | Kan^R^, p15A, P*_trc_*-OsNOMT-T*_rrnB1_* | This work |
| SBC008376 | Kan^R^, SC101, P*_lacUV5_*-pheA(G309C)-ppsA-aroF(P148L)-tktA(G369C) | *^15^* |
| SBC010507 | Kan^R^, ColE1, P*_lacUV5_*-AtCHI-T_B1006_-P*_trc_*-Gm4CL-T_B1006_-P*_trc_*-CnCHS-AtPAL-T*_rrnB1_* | *^16^* |
| SBC012916 | pSIM*cpf1* | *^14^* |
| SBC012918 | pTF-lacZ-rfp | *^14^* |
| SBC014335 | Kan^R^, p15A, P*_trc_*-AtF3H-T*_rrnB1_* | This work |
| SBC014337 | Kan^R^, p15A, P*_trc_*-AtFLS1-T*_rrnB1_* | This work |
| SBC015610 | Chl^R^, ColE1, P*_tet_*-GmF3H-T*_rrnB1_* | *^17^* |
| SBC015612 | Chl^R^, ColE1, P*_tet_*-OsF3H1-T*_rrnB1_* | *^17^* |
| SBC015614 | Chl^R^, ColE1, P*_tet_*-PhF3H-T*_rrnB1_* | *^17^* |
| SBC015616 | Chl^R^, ColE1, P*_tet_*-CsF3H-T*_rrnB1_* | *^17^* |
| SBC015618 | Chl^R^, ColE1, P*_tet_*-CuFLS-T*_rrnB1_* | *^17^* |
| SBC015673 | Amp^R^, p15A, P*_trc_*-SbFNSII-AtCPR-T*_rrnB1_* | *^17^* |
| SBC015674 | Amp^R^, p15A, P*_trc_*-ObFNSII-AtCPR-T*_rrnB1_* | *^17^* |
| SBC015675 | Amp^R^, p15A, P*_trc_*-GmFNSII-AtCPR-T*_rrnB1_* | *^17^* |
| SBC015676 | Amp^R^, p15A, P*_trc_*-GhFNSII-AtCPR-T*_rrnB1_* | *^17^* |
| SBC015677 | Amp^R^, p15A, P*_trc_*-LjFNSII-AtCPR-T*_rrnB1_* | *^17^* |
| SBC015883 | Amp^R^, p15A, P*_trc_*-GmF3H-CuFLS-T*_rrnB1_* | *^17^* |
| SBC015884 | Amp^R^, p15A, P*_trc_*-GmF3H-T_B1006_-P*_trc_*-CuFLS-T*_rrnB1_* | *^17^* |
| SBC015885 | Amp^R^, p15A, P*_trc_*-GmF3H-T_B1006_-P*_lacUV5_*-CuFLS-T*_rrnB1_* | *^17^* |
| SBC015886 | Amp^R^, p15A, P*_lacUV5_*-GmF3H-CuFLS-T*_rrnB1_* | *^17^* |
| SBC015887 | Amp^R^, p15A, P*_lacUV5_*-GmF3H-T_B1006_-P*_trc_*-CuFLS-T*_rrnB1_* | *^17^* |
| SBC015888 | Amp^R^, p15A, P*_lacUV5_*-GmF3H-T_B1006_-P*_lacUV5_*-CuFLS-T*_rrnB1_* | *^17^* |
| SBC015889 | Amp^R^, p15A, P*_trc_*-CuFLS-GmF3H-T*_rrnB1_* | *^17^* |
| SBC015890 | Amp^R^, p15A, P*_trc_*-CuFLS-T_B1006_-P*_trc_*-GmF3H-T*_rrnB1_* | *^17^* |
| SBC015891 | Amp^R^, p15A, P*_trc_*-CuFLS-T_B1006_-P*_lacUV5_*-GmF3H-T*_rrnB1_* | *^17^* |
| SBC015892 | Amp^R^, p15A, P*_lacUV5_*-CuFLS-GmF3H-T*_rrnB1_* | *^17^* |
| SBC015893 | Amp^R^, p15A, P*_lacUV5_*-CuFLS-T_B1006_-P*_trc_*-GmF3H-T*_rrnB1_* | *^17^* |
| SBC015894 | Amp^R^, p15A, P*_lacUV5_*-CuFLS-T_B1006_-P*_lacUV5_*-GmF3H-T*_rrnB1_* | *^17^* |
| SBC015959 | Kan^R^, p15A, P*_trc_*-PcFNSI-T*_rrnB1_* | This work |
| SBC015961 | Kan^R^, p15A, P*_trc_*-PaFNSI-T*_rrnB1_* | This work |
| SBC015964 | Kan^R^, p15A, P*_trc_*-CcFNSI-T*_rrnB1_* | This work |
| SBC015967 | Kan^R^, p15A, P*_trc_*-AcFNSI-T*_rrnB1_* | This work |
| SBC015970 | Kan^R^, p15A, P*_trc_*-AgFNSI-T*_rrnB1_* | This work |
| SBC015973 | Kan^R^, p15A, P*_trc_*-DcFNSI-T*_rrnB1_* | This work |
| SBC015976 | Kan^R^, p15A, P*_trc_*-AaFNSI-T*_rrnB1_* | This work |
| SBC015979 | Kan^R^, p15A, P*_trc_*-CmFNSI-T*_rrnB1_* | This work |
| SBC016066 | pTF-*yjiP*_*yjiR*::P*_trc_*-CgAccBC-CgAccD1-CgAccE | This work |
| SBC016090 | Chl^R^, ColE1, P*_lacUV5_*-AtCHI-T_B1006_-P*_trc_*-Gm4CL-T_B1006_-P*_trc_*-CnCHS-AtPAL-T*_rrnB1_* | This work |
| SBC016091 | Amp^R^, ColE1, P*_lacUV5_*-AtCHI-T_B1006_-P*_trc_*-Gm4CL-T_B1006_-P*_trc_*-CnCHS-AtPAL-T*_rrnB1_* | This work |
| SBC016092 | Chl^R^, p15A, P*_trc_*-AtF3H-T*_rrnB1_* | This work |
| SBC016093 | Chl^R^, p15A, P*_trc_*-AtFLS1-T*_rrnB1_* | This work |
| SBC016094 | Chl^R^, p15A, P*_trc_*-GmF3H-T*_rrnB1_* | This work |
| SBC016095 | Chl^R^, p15A, P*_trc_*-OsF3H1-T*_rrnB1_* | This work |
| SBC016096 | Chl^R^, p15A, P*_trc_*-PhF3H-T*_rrnB1_* | This work |
| SBC016097 | Chl^R^, p15A, P*_trc_*-CsF3H-T*_rrnB1_* | This work |
| SBC016098 | Chl^R^, p15A, P*_trc_*-CuFLS-T*_rrnB1_* | This work |
| SBC016130 | pTF-lacZ::lacI-P*_lacUV5_*-pheA(G309C)-ppsA-aroF(P148L)-tktA(G369C) | This work |
| SBC016187 | Kan^R^, p15A, P*_trc_*-ObFOMT1-T*_rrnB1_* | This work |
| SBC016189 | Kan^R^, p15A, P*_trc_*-ObFOMT2-T*_rrnB1_* | This work |
| SBC016191 | Kan^R^, p15A, P*_trc_*-MpOMT1A-T*_rrnB1_* | This work |
| SBC016193 | Kan^R^, p15A, P*_trc_*-EnOMT1-T*_rrnB1_* | This work |

**Supplementary Table S7.** MS parameters.

| Compound | Ionisation mode | Parent m/z | Daughter m/z | Dwell time [s] | Cone voltage [V] | Collision energy [eV] |
| --- | --- | --- | --- | --- | --- | --- |
| Chrysin | Positive | 255.0260 | 152.9334 | 0.015 | 4 | 34 |
| Galangin | Positive | 270.9528 | 104.9836 | 0.015 | 54 | 34 |
| Pinobanksin | Positive | 272.9727 | 148.9353 | 0.015 | 26 | 18 |
| Pinocembrin | Negative | 255.1543 | 213.1080 | 0.015 | 4 | 20 |
| Pinostrobin | Positive | 270.99 | 103.02 | 0.015 | 32 | 32 |

# References

[1] Shimizu, T., Lin, F., Hasegawa, M., Okada, K., Nojiri, H., and Yamane, H. (2012) Purification and identification of naringenin 7-*O*-methyltransferase, a key enzyme in biosynthesis of flavonoid phytoalexin sakuranetin in rice, *J. Biol. Chem.* *287*, 19315-19325.

[2] Berim, A., Hyatt, D. C., and Gang, D. R. (2012) A set of regioselective *O*-methyltransferases gives rise to the complex pattern of methoxylated flavones in sweet basil, *Plant Physiol.* *160*, 1052-1069.

[3] Willits, M. G., Giovanni, M. t., Prata, R. T., Kramer, C. M., De Luca, V., Steffens, J. C., and Graser, G. (2004) Bio-fermentation of modified flavonoids: an example of in vivo diversification of secondary metabolites, *Phytochemistry* *65*, 31-41.

[4] Somaletha Chandran, K., Humphries, J., Goodger, J. Q., and Woodrow, I. E. (2022) Molecular characterisation of flavanone *O*-methylation in *Eucalyptus*, *Int. J. Mol. Sci.* *23*, 3190.

[5] Pelletier, M. K., and Shirley, B. W. (1996) Analysis of flavanone 3-hydroxylase in Arabidopsis seedlings (Coordinate regulation with chalcone synthase and chalcone isomerase), *Plant Physiol.* *111*, 339-345.

[6] Wisman, E., Hartmann, U., Sagasser, M., Baumann, E., Palme, K., Hahlbrock, K., Saedler, H., and Weisshaar, B. (1998) Knock-out mutants from an *En-1* mutagenized *Arabidopsis thaliana* population generate phenylpropanoid biosynthesis phenotypes, *Proc. Natl. Acad. Sci. U. S. A.* *95*, 12432-12437.

[7] Martens, S., Forkmann, G., Britsch, L., Wellmann, F., Matern, U., and Lukačin, R. (2003) Divergent evolution of flavonoid 2-oxoglutarate-dependent dioxygenases in parsley, *FEBS Lett.* *544*, 93-98.

[8] Han, X.-J., Wu, Y.-F., Gao, S., Yu, H.-N., Xu, R.-X., Lou, H.-X., and Cheng, A.-X. (2014) Functional characterization of a *Plagiochasma appendiculatum* flavone synthase I showing flavanone 2-hydroxylase activity, *FEBS Lett.* *588*, 2307-2314.

[9] Gebhardt, Y. H., Witte, S., Steuber, H., Matern, U., and Martens, S. (2007) Evolution of Flavone Synthase I from Parsley Flavanone 3*β*-Hydroxylase by Site-Directed Mutagenesis, *Plant Physiol.* *144*, 1442-1454.

[10] Gebhardt, Y., Witte, S., Forkmann, G., Lukačin, R., Matern, U., and Martens, S. (2005) Molecular evolution of flavonoid dioxygenases in the family Apiaceae, *Phytochemistry* *66*, 1273-1284.

[11] Lee, T. S., Krupa, R. A., Zhang, F., Hajimorad, M., Holtz, W. J., Prasad, N., Lee, S. K., and Keasling, J. D. (2011) BglBrick vectors and datasheets: a synthetic biology platform for gene expression, *J. Biol. Eng.* *5*, 1-14.

[12] Jiang, Y., Chen, B., Duan, C., Sun, B., Yang, J., and Yang, S. (2015) Multigene editing in the *Escherichia coli* genome via the CRISPR-Cas9 system, *Appl. Environ. Microbiol.* *81*, 2506-2514.

[13] Hanko, E. K., Valdehuesa, K. N. G., Verhagen, K. J., Chromy, J., Stoney, R. A., Chua, J., Yan, C., Roubos, J. A., Schmitz, J., and Breitling, R. (2023) Carboxylic acid reductase-dependent biosynthesis of eugenol and related allylphenols, *Microb. Cell Fact.* *22*.

[14] Jervis, A. J., Hanko, E. K., Dunstan, M. S., Robinson, C. J., Takano, E., and Scrutton, N. S. (2021) A plasmid toolset for CRISPR‐mediated genome editing and CRISPRi gene regulation in *Escherichia coli*, *Microb. Biotechnol.* *14*, 1120-1129.

[15] Robinson, C. J., Carbonell, P., Jervis, A. J., Yan, C., Hollywood, K. A., Dunstan, M. S., Currin, A., Swainston, N., Spiess, R., Taylor, S., Mulherin, P., Parker, S., Rowe, W., Matthews, N. E., Malone, K. J., Le Feuvre, R., Shapira, P., Barran, P., Turner, N. J., Micklefield, J., Breitling, R., Takano, E., and Scrutton, N. S. (2020) Rapid prototyping of microbial production strains for the biomanufacture of potential materials monomers, *Metab. Eng.* *60*, 168-182.

[16] Dunstan, M. S., Robinson, C. J., Jervis, A. J., Yan, C., Carbonell, P., Hollywood, K. A., Currin, A., Swainston, N., Feuvre, R. L., and Micklefield, J. (2020) Engineering *Escherichia coli* towards de novo production of gatekeeper (2*S*)-flavanones: naringenin, pinocembrin, eriodictyol and homoeriodictyol, *Synth. Biol.* *5*, ysaa012.

[17] Yiakoumetti, A., Hanko, E. K., Zou, Y., Chua, J., Chromy, J., Stoney, R. A., Valdehuesa, K. N. G., Connolly, J. A., Yan, C., Hollywood, K. A., Takano, E., and Breitling, R. (2023) Expanding flavone and flavonol production capabilities in *Escherichia coli*, *Front. Bioeng. Biotechnol.* *11*, 1275651.
